# Advances in passively driven microfluidics and lab-on-chip devices: a comprehensive literature review and patent analysis

**DOI:** 10.1039/d0ra00263a

**Published:** 2020-03-23

**Authors:** Vigneswaran Narayanamurthy, Z. E. Jeroish, K. S. Bhuvaneshwari, Pouriya Bayat, R. Premkumar, Fahmi Samsuri, Mashitah M. Yusoff

**Affiliations:** Department of Electronics and Computer Engineering Technology, Faculty of Electrical and Electronic Engineering Technology, Universiti Teknikal Malaysia Melaka Hang Tuah Jaya 76100 Durian Tunggal Melaka Malaysia vigjes@gmail.com vigneswaran@utem.edu.my; InnoFuTech No: 42/12, 7th Street, Vallalar Nagar Chennai Tamil Nadu 600072 India; Centre of Excellence for Advanced Research in Fluid Flow, University Malaysia Pahang Kuantan 26300 Malaysia; Department of Biomedical Engineering, Rajalakshmi Engineering College Chennai 602105 India; Faculty of Electrical and Electronics Engineering, University Malaysia Pahang Pekan 26600 Malaysia; Faculty of Electronics and Computer Engineering, Universiti Teknikal Malaysia Melaka Hang Tuah Jaya 76100 Durian Tunggal Melaka Malaysia; Department of Bioengineering, McGill University Montreal QC Canada H3A 0E9; Faculty of Industrial Sciences and Technology, University Malaysia Pahang Kuantan 26300 Malaysia

## Abstract

The development of passively driven microfluidic labs on chips has been increasing over the years. In the passive approach, the microfluids are usually driven and operated without any external actuators, fields, or power sources. Passive microfluidic techniques adopt osmosis, capillary action, surface tension, pressure, gravity-driven flow, hydrostatic flow, and vacuums to achieve fluid flow. There is a great need to explore labs on chips that are rapid, compact, portable, and easy to use. The evolution of these techniques is essential to meet current needs. Researchers have highlighted the vast potential in the field that needs to be explored to develop rapid passive labs on chips to suit market/researcher demands. A comprehensive review, along with patent analysis, is presented here, listing the latest advances in passive microfluidic techniques, along with the related mechanisms and applications.

## Abstract 2

Different approaches employed in the passively driven microfluidics and LOC devices.

## Graphical abstract

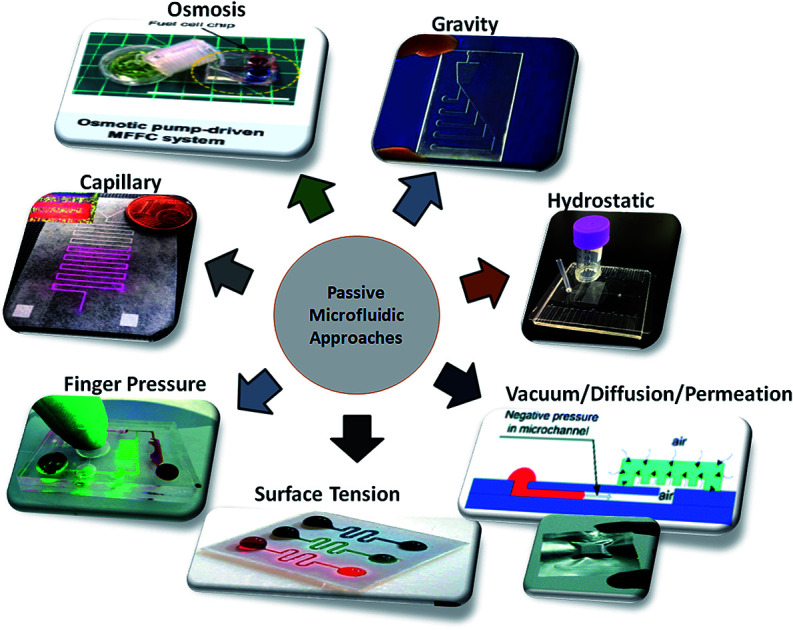

## Introduction

Handling small volumes of fluids is very important in high-throughput screening, diagnosis, and research applications.^1^ Microfluidics is one way to handle small volumes of fluids between microlitres (10^−6^) and picolitres (10^−12^).^2–4^ Hundreds of simultaneous biochemical reactions can be performed in a collection of microarrays arranged on a solid substrate which acts as laboratories, embedded in which are chips known as biochips.^5,6^ There are three main types of biochips: lab on chips (LOCs), DNA chips, and protein chips. LOCs employ a combination of one or more laboratory functions within a single integrated chip.^7^ Some fields utilizing LOCs, such as sub-micrometer and nano-sized channels, DNA labyrinths, single-cell detection and analysis,^8^ and nano-sensors, might become feasible, allowing new ways to interact with biological species and large molecules. In addition, a large number of biochemical analyses can be screened at a faster rate in disease diagnosis and the detection of bioterrorism agents.^9^ Several reports have been published on the various aspects of these devices, including fluid transport, system properties, sensing techniques,^10^ and bioanalytical applications. Advantages^11,12^ include lower fabrication costs, allowing cost-effective disposable chips and mass production. Simple tests that could be performed by the bedside are known as point-of-care (POC) testing.^13^ The ultimate aim of this technique is to obtain results in a concise period at or near the location of the patient, so that the treatment plan can be adjusted.^14^ Microfluidics can be used for various lab experiments, such as drug testing and discovery,^15–23^ filtration and separation of particles,^24^ cell sorting and counting,^25–31^ cell culture,^32–40^ point-of-care diagnosis,^41,42^ 3D printing, stoichiometry, and flow synthesis.^43,44^ Due to their simplicity with high throughput and very low reagent consumption,^45^ microfluidic chips are vital components in research, for the delivery of accurate results.^46^ Microfluidic chips are mostly made up of PDMS (polydimethylsiloxane). PDMS is commonly used because it is a transparent elastic polymer, permeable to oxygen and carbon dioxide.^47–51^ Additionally, PDMS is now becoming a standard material as it can be easily fabricated for microfluidic devices (MFDs), and its high gas solubility, which obeys Henry's law, is a significant advantage of using PDMS material.^52^

### Microfluidic operation techniques

The techniques assisting fluid flow in an MFD are generally classified as active or passive.^53,54^ Active microfluidics^55,56^ involves the movement or transport of biological samples and analysis of those samples through an external power source/field^57–59^ or actuators,^60^ such as peristaltic pumps,^61^ electro-kinetics,^62,63^ electro-wetting,^64^ electro-osmotic pumps,^65^ electrostatics,^66,67^ centrifugal and magnetic pumps^68^ and some other large power sources to power the pumps and actuators.^69–71^ Thus the complexity of structure and size is increased, which requires additional human resources. Hence, the probability of integrating active microfluidics with LOCs and (POC) applications has dropped off. To counter these drawbacks,^72–74^ the movement of the test sample is achieved either using fluid properties or passive mechanisms without any external supporting power sources. Hence, passive microfluidics^75^ has been adopted and used to a large extent in modern-day research projects to avoid the use of external supporting power devices. This method is simple, easy to manufacture, and does not need any actuators or external power supplies, as it employs basic laboratory instruments, like micropipettes, and medical devices, such as syringe pumps.

## Systematic literature search

**Fig. 1:**
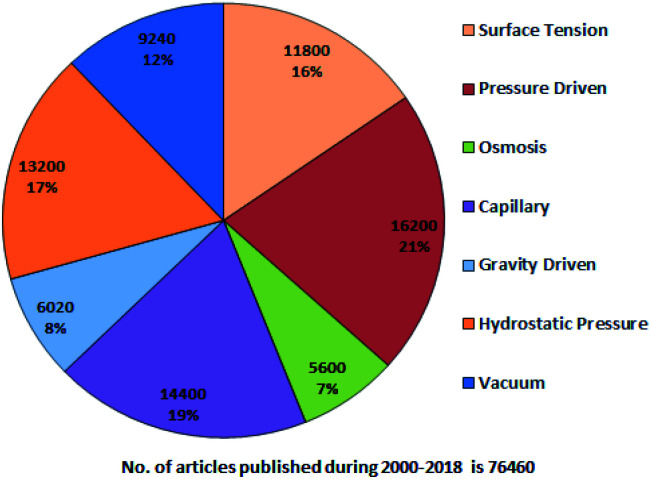
Trends in passive microfluidics.

A systematic literature search has been performed using Google Scholar with the following keywords: “passive pumping platform, passively driven microfluidics, pressure head driven microfluidics, and flow-driven microfluidics” throughout 2000–2018. Fig. 1 presents a pie chart revealing the aggregate number of articles on passive microfluidics published from 2000 to 2018. This review article will be useful for researchers who plan further investigations in this field.

From Fig. 1, it is evident that pressure-driven, capillary, hydrostatic, surface-tension and vacuum-based methods are leading trends. From the search, articles were selected and some were hand picked based on their relevancy with an exclusive focus on the passively driven approach and they are presented according to their timeline. In 2002, David Beebe *et al.* published a short report on the fabrication of MFDs and the physics applied to passive valves, mixers, and pumps which facilitate fluid flow.^53^ Due to the extensive usage of PDMS in MFDs, Sia *et al.* reviewed the advantages of using PDMS in miniaturized biological assay devices, such as efficiency and spectral insights into cell biology, where the fluidic flow was obtained by applying a positive or negative pressure at the inlet or outlet, respectively.^76^ Subsequently, Bayraktar *et al.* reviewed the available knowledge based on the areas that required additional investigation in pressure-driven and electro-osmotic flows in microchannels.^77^ Later, Fiorini *et al.* in 2005 reported a review of different modes of on-chip operations, such as the pumping and valving of fluid flow; and the separation and detection of different chemical species that have been implemented in a microfluidic format.^54^ Haeberle *et al.* reported platforms for LOC applications.^78^ Eventually, a microfluidic cell culture was developed, which implied the use of surface tension, where a differential pressure was generated due to the different volumes in the inlet and outlet port to assist the fluid flow and which was based on concepts related to physical and microenvironments on passive pumping.^79^ Additionally, Ahn *et al.* reported a short review in 2010 based on various methods of passive pumping and their applications to LOC biochemical analysis.^80^

Furthermore, Gervais *et al.* focused on various techniques that have been adapted for passive pumping.^74^ Later, Su *et al.* reviewed the latest advances in microfluidic platforms for POC testing in the context of infectious diseases, along with the integration of multiple functions into a single unit with full automation and analysed the challenges involved.^81^ Subsequently, Byun *et al.* summarized recent advances in pumping techniques for microfluidic cell culture in an effort to support current and potential users of microfluidic-based devices for advanced *in vitro* cellular studies.^57^ The relevant biophysical laws, along with their experimental details and the designs of various passive separation techniques, were explained by Tripathi *et al.* in 2015.^82^ These separation techniques advanced the development of single-cell capture. Eventually, Narayanamurthy *et al.* focussed on the development of single-cell trapping using hydrodynamic effects for the purpose of developments in cell separation and rapid viral detection and explained its benefits.^83^

In this article, we wish to discuss the current proposals, developments, updates, and future of passive microfluidic and LOC devices. The different methods adopted in every passively driven type of microfluidics are briefly covered, so that one can obtain a better and clearer knowledge of previous techniques.^84–86^ This also throws light on the advantages, flow rates and applications of all the techniques. The limitations of each method are also explained.

### Patent analysis and current key market players in the field of microfluidics and lab-on-chip devices

**Fig. 2:**
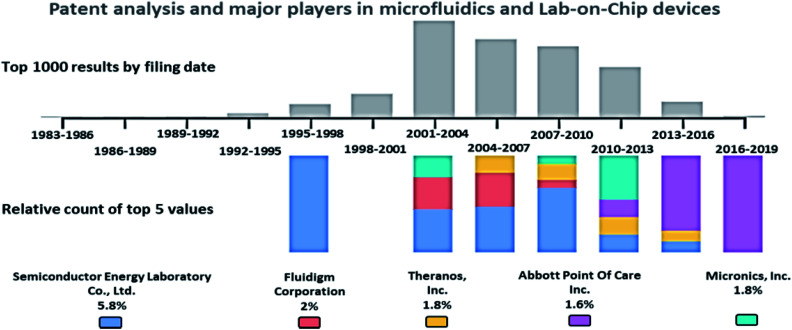
Patent analysis of passively driven microfluidics and LOC devices.

A patent analysis was performed with the Google Patents search tool, using the keywords, passive AND (driven OR flow) AND (biochips OR microfluidics OR LOC), and the analysis is provided in Fig. 2. The key players in the microfluidics market are Semiconductor Energy Laboratory Co., Ltd. (US), Theranos, Inc. (US), Abbott Point Of Care Inc. (US), Micronics, Inc. (US), Danaher (US), Thermo Fisher (US), PerkinElmer (US), Roche (Switzerland), Biomicro Systems, Inc. (US), Incyte Genomics, Inc. (US), Silicon Laboratories, Inc. (US), Tecan Trading Ag (Switzerland), Aviva Biosciences Corporation (US), Eksigent Technologies, Llc (US), Nanostream, Inc. (US), International Business Machines Corporation (US), 10X Genomics (US), Accelerate Diagnostics, Inc. (US), Advanced Cell Diagnostics, Inc. (US), Affymetrix, Inc. (US), Agilent Technologies, Inc. (US), Amphasys AG (Switzerland), Angle Plc (UK), Beckman Coulter, Inc. (US), Becton, Dickinson, and Company (US), Bio-Rad Laboratories, Inc. (US), Clontech Laboratories, Inc. (US), Celsee Diagnostics (US), Enumeral (US), Epic Sciences (US), Fluidigm (US), Illumina, Inc. (US), Kellbenx Inc. (US), Merck KGaA (Germany), NanoString technologies (US), Qiagen NV (Netherlands), Sigma Aldrich (US), Thermo Fisher Scientific, Inc. (US), Wafergen Bio-Systems, Inc. (US), Yikon Genomics (China), Zephyrus Biosciences, Inc. (US), Dolomite Microfluidics (UK), GYROS PROTEIN TECHNOLOGIES AB (Sweden), Sphere Fluidics (UK), OPKO Health (US), Waters (US), thinXXS Microtechnology (Germany), Abaxis (US), bioMérieux (France), Dolomite Microfluidics (UK), Microfluidic ChipShop (Germany), Elveflow (France), Cellix (Ireland), Micronit Microtechnologies (Netherlands), MicroLiquid (Spain), MiniFAB (Australia), uFluidix (Canada), Micralyne (US), and Fluigent (France).^87^

### Passive microfluidic techniques

Passive microfluidics is an emerging technique with a tremendous scope that is being adopted more commonly in LOC and POC diagnosis due to the following characteristics:

• Easy fabrication: Fabrication of passive microfluidics is simple and easy, as it avoids complicated fabrication resulting in complex structures.

• Less expertise: Passive microfluidics operation does not usually require training or experience, as it is straightforward and easy to operate.

• No external power source required: Passive microfluidics does not involve any external power source for its working.

• Low cost: Passive microfluidics is substantially low in price due to less complicated procedures and no auxiliaries being involved.

• Compact and portable: Passive microfluidics is very compact and portable in size; hence, it can be driven almost anywhere.

**Fig. 3:**
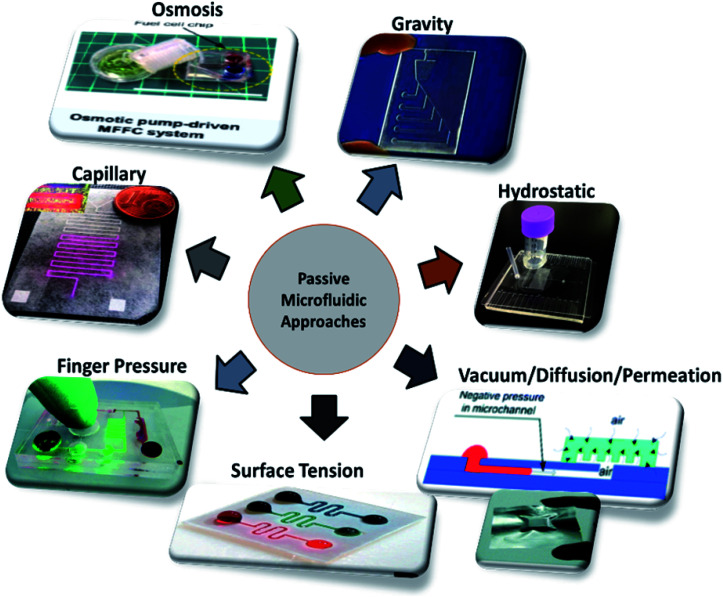
Different approaches employed in passively driven microfluidics and LOC devices.

Different techniques employed in the field of microfluidics and LOC devices to achieve passive operations are surface tension, pressure-driven, osmosis, capillary action, gravity-induced flow, vacuum suction, and hydrostatic pressure, as shown in Fig. 3. Each technique has its pros and cons. In the sections below, each technique is described in detail, and the articles are listed in year order.

### Surface tension

In general, surface tension is defined as a property of the surface of a liquid that allows it to resist an external force due to the cohesive nature of water molecules. This cohesive forces between the liquid molecules are responsible for the phenomenon known as surface tension.^88^

In microfluidics, the generation of strong passive flows can be achieved by selecting a suitable surface and liquid combinations that can create the required solid–liquid surface-tension gradients.^89^ Mathematical models are required to finalize the design of the channel, so Makhijani *et al.* developed a numerical model to simulate the liquid filling due to the presence of surface tension in the liquid–air interface and demonstrated the application of disposable biochips for clinical diagnosis. It was mainly used for analysis and optimization to achieve the desired flow.^90^ Subsequently, Walker *et al.* proposed a simple, semi-autonomous method of pumping fluids where devices producing small droplets, such as a pipettes, were required.^91^ Meanwhile, evaporation occurred during the surface-tension-based flow, which could be used to increase the concentration of the sample in the channel.^92^ Due to the requirement of microfluid mixing, for the purpose of drug delivery and research, Chen *et al.* demonstrated a micromixing device without the use of any active devices; hence, the surface tension of the fluid provided transport, merging, mixing, and stopping in the microchannel by varying the channel geometry.^93^ Ward *et al.* analysed droplet formation using flow-focusing geometry and microfluidic technology and compared the two methods of supplying fluids using a syringe-pump methodology that dictated the volume flow rates and a method which controlled the inlet fluid pressure.^94^ Later, Tan *et al.* controlled the droplets by the formation of a bifurcated junction in an MFD, and thereby controlled the chemical concentration in the droplet by the use of droplet fission.^95^

Furthermore, a passive pumping technique was employed in a direct methanol fuel cell (DMFC), through passive fuel delivery, designed based on a surface-tension driving mechanism and integrated with a laboratory-made prototype to achieve fixed consumption depending on fuel concentration and power-free fuel delivery. Due to different surface-tension properties, water was separated from methanol through a Teflon membrane, and forward flow occurred in the capillary. This was believed by Yang *et al.* to be more applicable to future small-scale DMFCs in portable electronics.^96^ Thanks to their analysis of channel pressure and flow, Berthier *et al.* reported that greater pressure created a large drop in output at the channel exit when a small droplet was placed on the entrance of the microfluidic channel.^97^ Eventually, in 2008 Ivar *et al.* published an article that implied the use of surface tension to induce a pressure difference in the fluid that causes pumping, routing, and compartmentalization without the use of any additional micro-components. Moreover, they demonstrated the applications of patterning multiple monolayer cell colonies and three-dimensional cell compartments and co-cultures.^98^ Backflow is a serious issue faced in surface-tension-driven pumps, so Ju *et al.* explained the optimum conditions required to avoid backflow in surface-tension-driven passive pumping by determining the ratio of inlet and outlet pressure during the occurrence of backflow.^99^ Du *et al.* reported that the concentration gradient in the channel induced forward flow, and it was further enhanced by evaporation-induced backward flow. Thus, the gradient concentration generated was controlled by convection and molecular diffusion. This approach was particularly used for chemical and biological processes in portable MFDs, where long-range gradients are required.^100^ Jane *et al.* analysed the flow rate with the Hagen–Poiseuille equation, thermodynamics, and the Young–Laplace equation where the flow rates could be controlled by channel geometry, dimension of inlet and outlet wells and hydrophobicity. Later, Chen *et al.* provided a clear idea about the surface-tension-induced flow rates, theoretical relationships among sample volume, induced flow rate, and surface tension of the drops at the inlet and outlet ports, and resistance to flow.^101^

As soon as the droplet was placed at the inlet port, it began to collapse due to varying velocity in the channel and it transformed from a smaller drop to a larger drop. Theoretically, the maximum flow rate was obtained at a contact angle of 90 degrees, but practically it was maintained between 30 and 60 degrees due to refilling. Resto *et al.* provided a clear idea of both theoretical and practical aspects of the pumping and the contact angle to achieve the maximum flow rate.^102^ The surface tension caused a pressure difference in the channel to induce fluid flow from the inlet to the outlet. Equilibrium of pressure in the channel led to the immediate arrest of the inflow. This sudden stop allowed its use in a wide range of biological applications from reagent delivery to drug-cell studies.^103^ Jane *et al.* explained that the surface tension in the fluid induced the flow through the microchannels due to the change in volumes of fluid. Passive microfluidics with electrochemical sensors inside the microchannel was considered for LOC flow injection. Various factors affecting the flow in the microfluidic channel were also discussed. This was the first report on a passive pump based on flow injection analysis (FIA).^104^ In 2010, Amy *et al.* integrated particle counting with a passive pumping mechanism by placing a 0.5 microliter drop of saline and sample fluid on the focussing inlet and the sample inlet, respectively. The surface tension in the fluid experienced a flow due to the change in volume. These flows to the reservoir traversed a pore that caused a change in resistivity, and the pulse was counted. Thus, particle counting was achieved with the help of surface-tension pumping.^105^

Puccinelli *et al.* explained that the pumping process of a small drop placed at the inlet was due to the surface tension in the inlet fluid. They validated the performance of a complete, reliable, and repeatable cell-based biological assay. The robustness of each technique was also discussed.^106^ Chung *et al.* described the involvement of surface tension, as well as evaporation, leading to the generation of passive pumping in a forward and backward direction with a concentration gradient a few centimeters long in the channel. Recent developments in microfluidic gradient generators were also described in their work.^107^ Besides research, Lin *et al.* demonstrated the uses and applications of microfluidics in the fields of food, environment, and physiological health monitoring.^108^ Berthier *et al.* suggested that the surface tension of the fluid enabled a short-term laminar flow patterning in multiple fluids when the sample was loaded in any sequence. Numerical simulations and practical experiments were conducted to study the laminar behaviour. This method was well suited to a cell-based assay and reduced the complexities of laminar flow patterning (LPF).^109^ In 2012, Resto *et al.* developed inertia-enhanced passive pumping that reduced fluid exchange and inertia-activated flow, which initiates the flow in an empty channel where fluid flow took place due to surface tension. They also analysed the transfer of momentum to the incoming fluid and the effect which induced the pumping mechanism.^110^

**Fig. 4:**
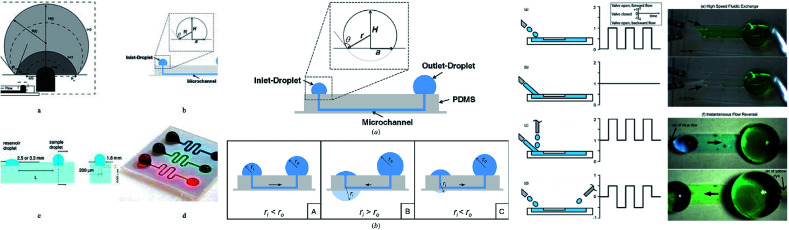
(i) The types of surface-tension-driven passive microfluidics in different studies, which include the recession of a drop in two phases along with a front view and side view of the microfluidic system in passive pumping (this figure has been adapted from ref. 113 with permission from ELSEVIER, copyright: 2018). (ii) A schematic diagram of surface tension driven microfluidics. The flow direction is basically determined by the radius balance of the droplets; the bold arrows in the figures denote the flow direction (this figure has been reproduced from ref. 99 with permission from IOP, copyright: 2008). (iii) Different surface-tension-driven flows: (a) pulsatile forward flow, (b) constant forward flow, (c) constant forward-flow with additional liquid packets, (d) alternating (forward/backward) flow, (e) whole-channel fluidic exchange achieved with alternating constant flow by two nozzles, and (f) instantaneous flow reversal using alternating (forward/backward) flow (this figure has been adapted from ref. 110 with permission from the Royal Society of Chemistry, copyright: 2012).

Microdevices are capable of targeted focal delivery of chemicals for axonal growth studies. Hence, Kuo *et al.* varied the drop volume to passively drive the flow into the microchannel. With this manipulation technique, the bio-chemicals delivered were combined with neuronal cells, and the required flow rate was achieved.^111^ Groot *et al.* enabled the dynamic culture and analysis of tissues in a hanging drop example, which employed surface tension as the driving force with two droplets, namely the culture droplet and the user-interface droplet.^112^ Computer simulations were used to give detailed information on flow patterns and physical phenomena under different conditions. The pumping process was divided into three planes with deceleration followed by acceleration and deceleration that relied on the physical properties of the operating fluid, and geometrical characteristics of the channel.^113^Fig. 4 shows an MFD utilizing surface tension for passive operation. The latest reports on passive MFD using surface tension techniques are summarised in Table 1.

**Table 1:** Recent surface-tension-driven passive pumping techniques in microfluidics

S. no	Analytes used	Materials used	Auxiliaries involved	Flow rate	Advantages	Disadvantages	Applications	Ref. no
1	Air and water	PDMS, SU-8 photoresist		10 μl min^−1^	Newtonian and non-Newtonian fluids were handled	A computational model was studied alone	Disposable microfluidic biochips	90
2	Water	PDMS	Pipette or syringe	1.25 μl s^−1^	Easy interfacing	Evaporation occurs	Small labs and high-throughput assaying systems	91
3	Fluorescent spheres in DI water	PDMS	Pipette		Constant flow rate	Evaporation is a slow process	Biological or chemical application	92
4	Red and green fluorescent spheres in DI water	PDMS	Pipette		Simple and portable	The mixing process begins only after the merging of liquids	Micro total analysis system	93
5	Pure oleic acid and water	PDMS moulds with glass	Syringe pump		Reduced use of reagent	Droplet symmetric is corrected only with the asymmetric flow	High-throughput screening	95
6	Pure methanol	PDMS		2 ml min^−1^		Tough transportation of methanol in a few hours	Small-scale DMFCs for portable electronics	96
7		PDMS, SU-8 photoresist	Micropipette		Practically analyzed and long lasting	Increase in the output pressure decreases flow	Cell studies	97
8	Cell culture medium with protein	PDMS	Pipette	34.6 nl s^−1^ to 16.6 μl s^−1^	Highly parallel arrays with 3D cell cultures are employed	Not applicable to high-density valve arrays	Diagnostics and drug development	98
9	Mixture of DI water & fluorescent particles	PDMS	Pipette			Backflow due to flow rotation of outlet liquid		99
10	DI water and methanol	PDMS	Pipette	85 nl s^−1^ to 196 nl s^−1^		Geometrical properties can affect the flow rate	LOC devices	101
11	DPBS with FITC dextran (culture medium)	PDMS	Pipette		Easily adaptable with high throughput	Gradient generation is affected by fluid viscosity	Biological and drug discovery applications	100
12		PDMS	Automated fluid delivery system	4 ml min^−1^	No substrate bonding is required	Flow rates are limited by device dimension	Cell culture and biological applications	102
13	Fluorescent beads	PDMS	Automated fluid delivery system	4 ml min^−1^	No substrate bonding is required	Control of flow direction is difficult	Biological applications, drug-cell studies	103
14	Blood or plasma mixed with glycerol	PDMS	Pipette		Minimal reagent consumption, and waste generation	Drastic decrease in flow rate over time	Immunoassays and LOC	104
15	Saline	PDMS	Pipette	2.35 μl min^−1^	Particle counting is done within the system	External power source is required for counting	POC or diagnostic MFD	105
16	Normal urine, mammary gland epithelial cells	PDMS	Pipette or automated liquid handling system		Reduced number of cells and reagents are required	Reagents with different viscosity or surface tension should be optimized	Screening in cell culture and biological cell-based assay	106
17	Neutrophils, tumor cells, stem cell, neurons, and bacterial cells	Glass substrate with PDMS mould	Pipette		Manipulation of fluid flow, and real-time monitoring of the cells	Complicated microfabrication process	Chemotaxis, stem cell differentiation, and endothelial cell migration	107
18		PDMS	Pipette		Low reagent consumption and easy to fabricate	Low efficiency	Food and remote military operations, home healthcare	108
19	PBS with red and green food colorant	PDMS	Pipette		Cell population can be patterned	Mixing of sample	Wound-healing assay	109
20	Green, yellow and blue dyes	PDMS		4 ml min^−1^	High flow rates are observed	Designing of small channel is tough	Cell-based assays	110
21	Axon, DI water	PDMS, parylene	Pipette	∼0.63 μl s^−1^	Flexible and user convenient	The same chemical concentration	Axonal guidance studies	111
22	Bone marrow stromal cells	Polymer sheets	Micropipette		Separation of cells is comparatively good	Increased space requirement	Microscale metabolomics	112
23	Water		Micropumps	4 ml min^−1^	Straight forward implementation of channels	Radii of the droplet vary	Drug delivery and cell biology	113

#### Limitations

As the fluid droplet at the inlet reduces, the surface tension decreases causing a decrease in fluid flow. So refilling is necessary for continuous flow.^57^ Additionally, the presence of reflected light and mirage effects in millimetre-wide spherical caps reduce the accuracy of goniometer measurement.^97^

### Pressure-driven

**Fig. 5:**
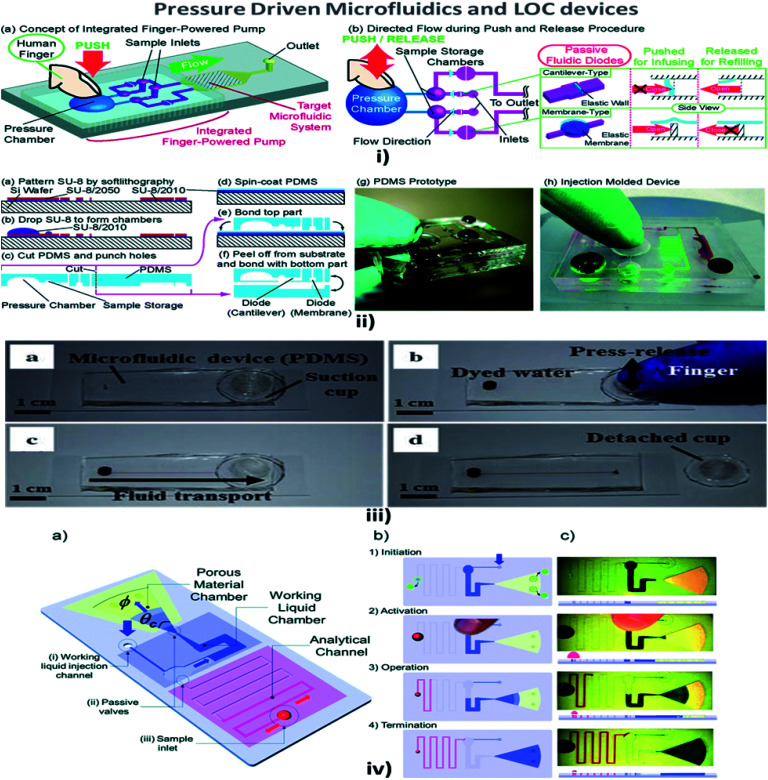
(i) (a) An integrated human-powered pumping system. By pushing the deformable chamber, mechanical pressure infuses the solution from the inlets to the outlets; (i) (b) the pumping procedure of the finger-powered pump with push-and-release actions. (ii) Fabrication *via* a multilayer soft lithography process; (ii) (a–f) the device consists of three layers of PDMS; (ii) (g) a fabricated PDMS pumping system; (ii) (h) a fabricated prototype *via* the injection moulding process (figures (i) and (ii) have been adapted from ref. 127 with permission from the Royal Society of Chemistry, copyright: 2014). (iii) Suction-cup-driven equipment-free fluid pumping: (a–d) finger-triggered pumping and reversible integration of the PDMS suction cup to the MFD (figure (iii) has been reproduced from ref. 132 with permission from SPRINGER, copyright: 2018). (iv) A self-powered imbibing microfluidic pump by liquid encapsulation (SIMPLE): (a) sequential pump operation and (b) an experimental presentation. (c) Initially, the chip is prefilled with the working liquid (blue) through the inlet denoted by 25 thick blue arrows and encapsulated by impermeable protective foil patches (green circles). Before activation, the foil is removed, the sample (red) is deposited over the inlet hole, and temporary finger force can activate the pump. When the working liquid touches the paper, the finger can be removed, and the pump is activated. The pump works until the working liquid saturates the paper or until all of the fluid has been sucked into the paper (figure (iv) has been adapted from ref. 129 with permission from the Royal Society of Chemistry, copyright: 2014).

Fluid pressure is a measurement of the force per unit area. Pressure in liquids is equally divided in all directions; therefore, if a force is applied to one point of the liquid, it will be transmitted to all other points within the liquid.^114^ MFD and LOC devices employing pressure for their passive operations are shown in Fig. 5. In a passive pressure-driven technique, the pressure created in the reservoirs to drive the sample is achieved either by pipetting or by a finger-force pressure.^115^ Liu *et al.* reported a twisted microchannel with chaotic advection that possessed high potential mixing even for fluids with a low Reynolds number.^116^ Ahn *et al.* suggested the fluid control technique and verified that the low-pressure drop in the fluid tended to maintain the flow without any complicated pumps.^117^ Also, Jeon *et al.* described the design and fabrication of passive valves and pumps, which used the pressure-driven mechanism instead of electro-osmotic pumps. The fabrication included aligning, stacking, and bonding of a patterned membrane.^118^ Rush *et al.* established a 2D serpentine channel with the flow of low-Reynolds-number Brownian solute particles.^119^ Later, Moorthy *et al.* developed an on-chip porous filter that separates minimal volumes of biological fluids in real-time applications and analysis.^120^ Also, Hu *et al.* investigated the relationship between the pressure drop in the rough channel and smooth channels and also the pressure drop due to a change in height. It was found that in a rough channel the pressure drop increased and when the channel height increased the pressure drop decreased.^121^

Chen *et al.* explained that a wavy-wall section incorporated within the microchannel developed a centimetre-long high concentration gradient by increasing the interfacial leads.^122^ Jiang *et al.* explained the push or pull in the sample fluid due to a negative or positive pressure that a syringe pump would create.^123^ Hattori *et al.* confirmed that fluid flow commenced only when the pressure was applied to the fluid by using a syringe pump or micropipette through an air vent filter.^124^ In addition, when a finger-powered pressure of about 3–4 kPa was applied at the inlet, sample movement occurred.^114^ In their investigations, Davey *et al.* recommended a system where the inlet and the outlet were a hydrophobic and a hydrophilic needle, respectively, and the channel was made of a hydrophilic region where the fluid from the inlet traversed the channel and reached the outlet.^125^ Tice *et al.* reported an electrostatic microvalve with passive components embedded in them, which was used to regulate the pressure in hydraulic control lines and actuate pressure-driven components.^126^

Finger-power integrated pumping systems had been utilized to eradicate the limitations produced by the use of external pumps. Pressure generated inside the pressure chamber played a vital role in determining the efficiency of finger-powered pumps.^127^ Thus the requirement for pre-evacuation of PDMS devices in a vacuum chamber was eliminated. Thereby, the design of a simple POC pumping method using a single-layer structure where the dead-end microfluidic channel was partly surrounded by an embedded microchamber, with a thin PDMS wall separating the dead-end channel and the embedded microchamber, was reported by Xu *et al.*^128^ But the working liquid created a reduced pressure in the analytical channel and induced sequential sample flow into the microfluidic circuits. Kokalj *et al.* reported simplified activation by fingertip pressure with no external power or control for a wide range of applications in POC diagnostic settings.^129^ Jeong *et al.* developed an on-chip microflow control technology with the ability to mimic *in vivo* conditions at an *in vitro* microscale for long-term tissue culture with a continuous flow rate. The passive flow was initiated using a siphon effect, and a yarn flow resistor to regulate the flow rate in the microchannel.^130^ Thanks to implantable drug delivery systems, a micropump for controlled, automated inner-ear drug delivery with the ultimate aim of producing a long-term implantable/wearable delivery system was investigated by Tandon *et al.*^131^ Currently accessible pressure-driven approaches in passively driven microfluidics are summarised in Table 2.

**Table 2:** Recent pressure-driven approaches in passively driven microfluidics

S. no	Analytes used	Materials used	Auxiliaries involved	Flow rate	Advantages	Disadvantages	Applications	Ref. no
1	Sodium hydroxide and ethyl alcohol	PDMS membrane	Syringe pump		Minimum chances of clogging, fouling, and loss of sample	Increased in the complexity of flow	Biochemistry analysis, drug delivery	116
2		PDMS		1 μl min^−1^	Flow rate can be tailored based on channel height	Low flow rate	Biochemical analysis systems	117
3	Plasma	PDMS			Used where the electro-osmotic pumping is not feasible	Miniaturization of Si valves was difficult	Disposable diagnostic and drug screening applications	118
4	Incompressible Newtonian fluid					Limited by their total length		119
5	Fluorescent-labeled beads, rabbit blood	Pre-polymer, glass	Pipette	10 and 20 μl min^−1^	Eliminates the need for centrifuging principle		Drug delivery, cell culture	120
6	Water					Pressure drop in the microchannel in high	LOC	121
7	Incompressible Newtonian liquids					Both active and passive mixing are required	Drug delivery, DNA hybridization	122
8	Microbeads, blood	PDMS	Syringe pump		Inexpensive, fast and sensitive for fluid flow	Difficult to integrate with POC	Immunoassay	123
9	Water with red-coloured dye	Glass slide			3–4 kPa pressure alone is required	External pressure is required	POC diagnostics	114
10	Viscous liquid	PDMS	Syringe pump	500 μl min^−1^	Highly stable		Detection of air-borne contaminants	125
11	Culture media	PDMS	Micropipette		Efficient, user-friendly		Drug discovery	124
12	Drug of interest	Polyamide	Drug loading pump	40 μl min^−1^	Requires no additional valves to assist the flow	Constant dosage	Cochlear drug delivery system	133
13	Rabbit blood	PDMS, parylene	Microneedle		Simple *in vivo* blood sampling process	Needs to be operated in pneumatic platforms	Biosensor chip	126
14	Green-dyed water	3-Layered PDMS	Pipette	3.75 times greater than water flow	Transportation of singular fluids is possible	Pressure varies due to the use of different fingers	Point of care and disposable biomedical equipment	127
15	Deionised water	PDMS	Syringe	0.089 to 4 nl s^−1^	Syringe supplies pressure source to drive		POC pumping system	128
16	Blue and red dyed water	PDMS	Syringe or pipette	0.07, 0.12 and 0.17 μl s^−1^	Combined with the hydrophobic polymer system to stay stable	Speed decreases with filling the channel	Point of care diagnosis	129
17	Endothelial cell (human)	PDMS	Pipette	10–100 ml h^−1^	Physiological levels of flow can be regulated	Dependency on height	Cell-based applications	130
18		Layers of PEI for the rigid structure	Electronic dose control	13–18 μl min^−1^	Increased efficiency within the system	Reduction in system pressure is tough	Hair-cell regeneration therapy	^131^
19	Umbilical vein endothelial cells	PDMS	Pipette	950 μl min^−1^	Easy distribution of pressure		Cell culture in drug discovery and point of care	134
20		PDMS with oxygen plasma treatment		1.42 ml min^−1^	Constant flow rate under the sinusoidally varying signal is seen		POC diagnostic devices	135
21	Microparticle stock solution with DEX	CAD design for channels	Pipette or syringe	1.55 μl min^−1^	Mono-dispersed droplet formation is achieved		Cell analysis, cell-based assays	136
22	Sterile sputum	PDMS	Micropipette	4 ml min^−1^	High recovery rate of leukocytes		Pulmonary diseases detection	137
23	Phosphate buffer	PDMS	Syringe pump	80 μl min^−1^	Rapid fluid mixing		Enzyme-based assay	138
24	Dyed deionized water and mineral oil	PDMS	Pressing and releasing cup	Increasing flow rate	Simple and effective. Fabrication of cup is easy	Diameter of the cup stops the flow	Resource-limited applications	132
25	Glycerol diluted in water	PVC and PMMA	Finger activation	0.5–150 μl min^−1^			Drug delivery applications	139
26	Ethyl alcohol, acetone	PDMS	Teflon tube to release air	30.56–33.98 μl min^−1^	Increased flexibility and independent		Immunoassays	140

Satoh *et al.* in 2016 reported multiple medium-circulation units on a single MFD that were driven only by two pneumatic pressure lines with three independent culture units, in which the cells were cultured under medium circulation flow. The authors stated that pneumatic pressure could be easily distributed to multiple wells in a reservoir with a common gas-phase space without any changes in tube connections.^134^ Zhang *et al.* developed a microfluidic passive flow regulator with an in-built five-layer structure valve for high-throughput flow-rate control in microfluidic environments by constructing a gas-driven flow system and analyzed the flow regulation.^135^ Moon *et al.* investigated a passive microfluidic flow-focusing method to generate water-in-water aqueous two-phase system (ATPS) droplets without the involvement of any external components. This was the first microfluidic technique that formed low interfacial tension ATPS droplets without applying external perturbations.^136^ Ryu *et al.* examined a closed-loop operation of inertial microfluidics, which could dissociate clinical airway secretions and isolate/enrich immune-related cells for *in vitro* downstream assays. They found their applications in clinical *in vitro* cell-based biological assays of various pulmonary diseases like acute respiratory distress syndrome, pneumonia, cystic fibrosis and bronchiectasis.^137^

Lee *et al.* also developed a negative pressure-driven fluid flow generated by a simple finger-triggered operation where the PDMS suction cup was placed at the outlet of the device and dispensed the fluid at the inlet.^132^ A new infusion-based simple method (ISIMPLE) for drug delivery into the skin was developed by Dosso *et al.* with a self-contained skin patch without any external driving source, where the expelled air increased the pressure due to the extreme flexibility of the design and manufacture. The ISIMPLE concept offered enormous opportunities for entirely autonomous, portable, and cost-effective LOC devices.^139^ Later, Liu *et al.* developed a PDMS pump that utilized the air released from the aerated PDMS to create a positive pressure in the MFD.^140^

#### Limitations

The application of external pressure using a finger might affect the accuracy and repeatability of the assays due to pressure difference, and multi-step diagnostic assays may not succeed *via* hand-powered mechanisms since several reagents/buffers need to be driven in a controlled manner.^141^

### Osmosis

**Fig. 6:**
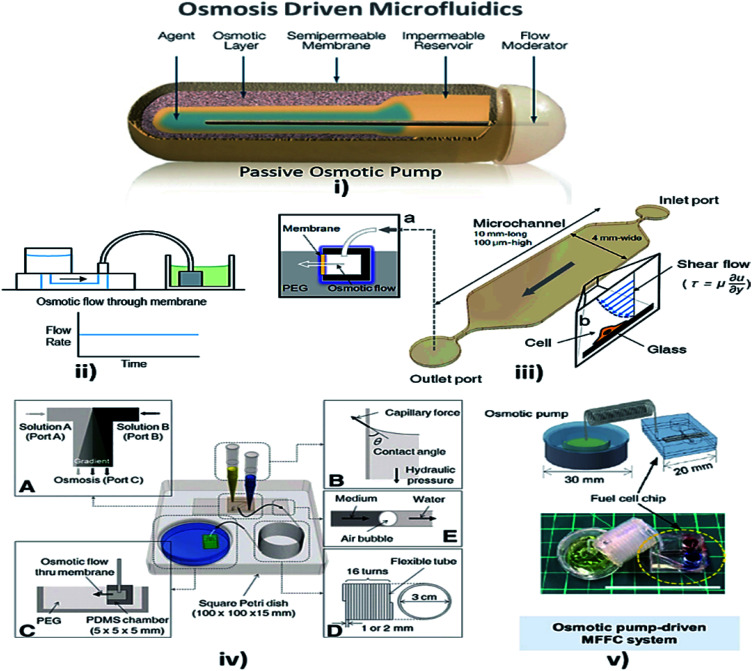
(i) Osmotic pump schematic diagram (this figure has been adapted from ref. 147 with permission from the Royal Society of Chemistry, copyright: 2009). (ii) Osmotic pump operation (this figure has been reproduced from ref. 57 with permission from Wiley, copyright: 2014). (iii) An operational diagram of the system, including the pump unit and the Poiseuille flow with constant wall shear stress (this figure has been adapted from ref. 148 with permission from IOS Press, copyright: 2010). (iv) Experimental setup with four components, namely concentration gradient, pipette tips, osmotic pumps and coiled tube (this figure has been reproduced from ref. 146 with permission from Alpha Med Press, copyright: 2009). (v) A microfluidic fuel cell (MFFC) system using an osmotic pump (this figure has been adapted from ref. 149 with permission from IOP, copyright: 2018).

Osmosis is the spontaneous net movement of solvent molecules to a region of higher solute concentration in the direction that tends to equalize the solute concentrations on both sides through a selectively permeable membrane. Permeability depends on solubility, charge, or chemistry, and solute size. Water molecules diffuse through the solute from the solvent layer.^142^ Bruhn *et al.* demonstrated the development of devices that are capable of pressure generation based on the osmosis principle, which could be made from available low-cost materials.^143^ Some MFD and LOC devices using osmosis as a passive approach for their operation are shown in Fig. 6.

Later, Good *et al.* integrated and analyzed a polymeric microfluidic device in a portable mechanical micro-pumping system that used fluid-responsive polymer particles as an actuator, without external power.^144^ In 2009, Xu *et al.* reported that, when a semi-permeable membrane was sandwiched between the inner osmotic reagent chamber and the outer water chamber, the water flow to the osmotic reagent chamber *via* the semi-permeable membrane facilitated the flow of fluid in the channel by the process of osmosis.^145^ Furthermore, stable concentration gradients are required for cell analysis and culture. The development of a microfluidic platform provided stable concentration gradients for cells of various signaling molecules for more than a week with only the least amount of handling and no external power source. Later, Park *et al.* optimized the osmotic pumping performance by balancing the capillary action and hydraulic pressure in the inlet reagent reservoirs.^146^ Updates on osmotic-driven passive pumping techniques in microfluidics are summarised in Table 3.

**Table 3:** Recent osmotic-driven passive pumping techniques in microfluidics

S. no	Analytes used	Materials used	Auxiliaries involved	Flow rate	Advantages	Disadvantages	Applications	Ref. no
1	Sieved particles	PDMS and natural rubber	Dropper	17 μl min^−1^	Can be placed directly on a microdevice	Reactivation of pump requires water	Finds the application in portable devices	144
2		PDMS chamber	Syringe	0.33 μl min^−1^	Low flow rate is used for constant refreshing of culture medium	Regular refreshing of osmotic reagent	Microfluidic flow injection system	145
3	Cholera toxin subunit B	PDMS cubic chambers	Micro-pipette	0.15 μl h^−1^	Provides the concentration gradient for more than a week	Low flow rate	Basic and translational research areas such as stem cell differentiation research and long-term cell culture	146

#### Limitations

An osmotically driven mechanism needs a complex setup compared to surface-tension, capillary, and gravity-driven setups.^57^ In osmotic drug delivery systems refilling the reservoirs was mostly impossible or complicated with the constant drug delivery rate.^144^ The development of an additional reservoir for the solvents increased the device complexity for operation in any environment.^150^

### Capillary

Capillary action is defined as the movement of a fluid within the spaces of a porous material due to the intermolecular forces between the liquid and the surrounding solid surfaces that enables the liquid to flow in narrow spaces without the assistance of, or even in opposition to, external forces like gravity.^87^

**Fig. 7:**
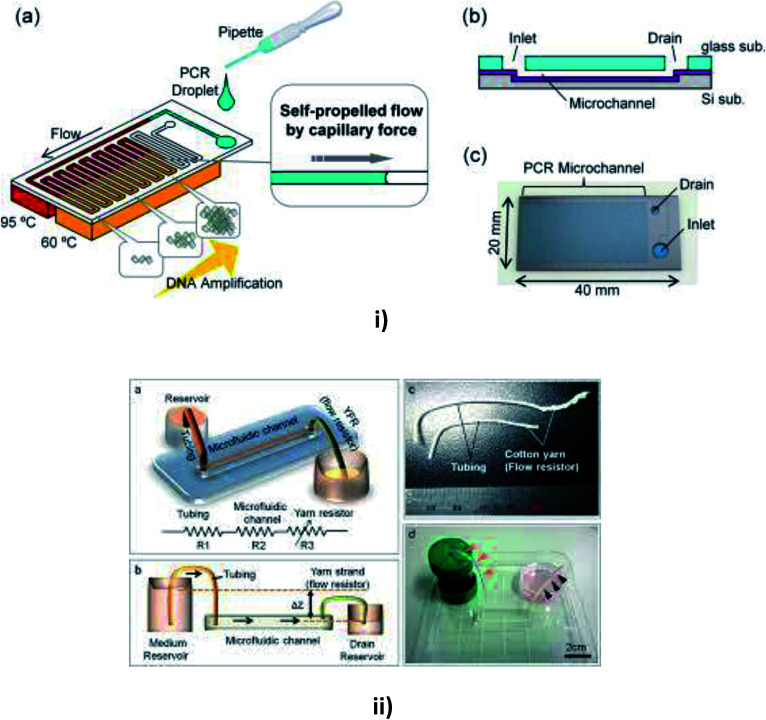
(i) Schematic diagrams of a microfluidic device for self-propelled continuous-flow PCR: (a) concept diagram; (b) a cross-sectional view of the microfluidic device. (c) A photograph of the fabricated microfluidic device (this figure has been reproduced from ref. 182 with permission from Elsevier, copyright: 2015). (ii) A yarn flow resistance (YFR)-regulated microflow control system: (a) a schematic diagram showing the siphon effects in the yarn-capillary-resistance-driven micropump system; (b) a schematic diagram of the water-head-difference-driven siphon effect controlled by the YFR; (c) a photographic image of a yarn capillary regulator; (d) the microfluidic device prepared with a YFR (this figure has been adapted from ref. 130 with permission from the Royal Society of Chemistry, copyright: 2014).

Juncker *et al.* reported a capillary pump assisted capillary flow microfluidic system, where the flow was activated from the open and closed channels of the device.^151^ MFD and LOC devices incorporating the capillary technique are shown in Fig. 7. Simultaneously, a microfluidic capillary system was presented for the continuous transport of fluid using capillary force, when the fluid was placed in the service port.^151^ Mixing of fluids is necessary for drug delivery and research. So, Hosokawa *et al.* reported an MFD which used capillary force for pumping.^152^ To avoid clogging by fluids, Kim *et al.* presented a capillary passive retarding microvalve at the junction of the microfluidic channel where the propagation occurred only after the merging of two fluids.^153^ An exact discussion of interface motion driven by capillary action in a microchannel was reported by Ichikawa *et al.* where a dimensionless variable of the driving force was used to predict the interface motion.^154^ In order to maintain and control the flow properties of capillary systems (CSs), Zimmermann *et al.* developed a design for capillary pumps. These capillary pumps were designed to have a small flow resistance and were preceded by a constricted microchannel, which caused flow resistance.^155^ Subsequently, Suk *et al.* developed a technique to control the autonomous capillary flow with a passive method where the flow could be retarded with appropriate hydrophobic patterns in hydrophilic channel surfaces. The microfluidic system was designed in such a way that it contained two planar parallel surfaces, separated by spacers.^156^

Flow rates may vary in different substrates, so Zhu *et al.* reported a study of capillary flow rate in several substances, including glass, polycarbonate (PC) and polydimethylsiloxane (PDMS) to measure the contact angles and to examine the longevity of capillary flow in PDMS and PC chips to give better clarity in measuring the flow rates.^157^ Lynn *et al.* realized a pressure difference arising from the small curved meniscus at the bottom of the outlet reservoir that drove the fluid with a constant fluid flow for more than an hour.^158^ Later, Gervais *et al.* described the fluid flow from high capillary pressure to low capillary pressure and increased the channel width at each level to reduce the friction, thereby leading to a high flow rate.^159^ Mukhopadhyay *et al.* proposed a microchannel bend in polymethylmethacrylate (PMMA) of different widths. The effects of channel aspect ratio and different separation angles were studied for fluid flow.^160^ Eventually, Alphonsus *et al.* explained in a short review on microfluidic immunoassay that the capillary pressure in the channel pushed the sample, causing the fluid to flow within the device.^161^ Kim *et al.* realized that the surfactant-added PDMS could be used to increase the hydrophobicity of PDMS to increase the filling rate of blood by capillary action.^162^ Souza *et al.* fabricated a toner-based microfluidic device in which the fluid flowed towards the outlet as soon as the serum was placed in the sample inlet.^163^ Later, Horiuchi *et al.* also developed an immunoassay chip which consisted of vertically integrated capillary tubes to create negative pressure and pump the fluid towards it.^164^ Once the fluid had been placed in the inlet well, Kim *et al.* verified the flow timing control and direction of multiple solutions in the channel.^165^

Kistrup *et al.* reported aspects of using rapid prototyping instead of pilot (mass) production. They included the fabrication of the microfluidic system that employed injection moulding and ultrasonic welding in which the fluid flow was assisted by the capillary pressure at the interface nozzle.^166^ Berthier *et al.* proposed the onset of the suspended capillary flows (SCF) and the viscous friction at the walls using the force balance between the capillary forces that drove the flow.^167^ Plasma separation is possible in passive pumping microfluidic technology. Madadi *et al.* demonstrated a self-driven blood plasma separation microfluidic chip, which was capable of extracting more than 0.1 μl of plasma from a single droplet of undiluted fresh human blood (5 μl) with high purity without any external pumps.^168^ Nie *et al.* reported a flexible microfluidic device that filled the channel through capillary force to provide continuous fluid pumping through an evaporation micropump. The hexagonal arrangement at the pore array drove the fluid flow and automatically absorbed liquid through a filter paper interface.^169^ Mukhopadhyay *et al.* described a leakage-free PMMA fabricated MFD with microchannel bends with the effect of surface wettability on surface-driven capillary flow. This type of microfluidic system was utilized in blood cell separation from whole blood.^170^ Maxime Huet *et al.* developed and integrated a biological protocol to observe RBC's agglutination in POC analysis. This work proved that the agglutination of blood began directly within the chip containing embedded reagents where a correlation-based indicator was used to conserve both spatial information and image quality.^171^ Later, Bunge *et al.* reviewed the utilization of symmetrical surface phase guides (SSPG), where the wall-less channels were generated by patterning hydrophobic phase guides on both sides of the chip to induce liquid propagation with the help of the capillary effect.^172^ Wu *et al.* generated flow-focusing droplets through the capillary effect to reduce the requirements of subsequent systems in addition to high flexibility.^173^

Zhai *et al.* developed a microfluidic device, which was insensitive to backflow due to the integration of a syringe pump to balance the capillary pressure within the channel.^174^ A study on the microfluidic self-flowing chip to understand the influence of micro-scale topographies was reported by Xie *et al.* According to this work, the flow rate increased with increasing gradient on the surface and the flow speed was 40 times greater with high efficiency.^175^ Vasilakis *et al.* reviewed a simple high-speed filling passive capillary pump integrated with lab-on-printed circuit board technology (Lo-PCB), with induced capillary pressure to produce stable flow rates.^176^ Akyazi *et al.* developed a new concept for fluid flow manipulation in micro paper-based analytical devices (PADs), where the ionogel (considered to be a negative passive pump) could drive fluids by the swelling effect, which controlled the flow direction and volume to the outlet.^177^ Frimat *et al.* described a new single-cell trapping procedure within the micro sieve electrode array (SEA) through capillary phenomena for an organized positioning of neurons to produce higher biocompatibility.^178^

Mei *et al.* developed a capillary-based open microfluidic device (COMD) for monodispersed droplet generation, from gas bubbles to highly viscous polymer solutions to provide high throughput in industrial emulsification. Capillary action was used as a portable sidewall of another microchannel with controllable size.^179^ Kim *et al.* described a liquid additive, which passively controlled the velocity of cells within a detectable range during capillary sample loading, thereby eliminating the need for bulky and expensive pumping equipment. It also required the adoption of an immune bead assay, which was quantified with a portable fluorescence cell counter based on a blue-light-emitting diode.^180^ Moonen *et al.* investigated capillary-based passive pumping for optimized neuronal cell trapping across a microsieve with gentle velocity profiles and high survival rates.^181^ Reports on capillary-based passive pumping in microfluidics are summarised in Table 4.

**Table 4:** Updates on capillary-based passive pumping in microfluidics

S. no	Analytes used	Materials used	Auxiliaries involved	Flow rate	Advantages	Disadvantages	Applications	Ref. no
1	Bovine serum albumin	Flat PDMS	Pipette	220 nl s^−1^	Free from dead volumes	Flow rate quickly reduces	Portable diagnostics, biological assays	151
2	Fluorescein ethanol	PDMS and glass surfaces	Syringe		Simple hardware setup and easy to operate	Depends on the contact angle for sample loading	Biological or chemical analysis	152
3	Deionised water	PDMS	Pipette		Clogging at the confluence is prevented	Liquid propagation is done only after the emergence		153
4	Ethanol	PDMS and Pyrex glass tube	Syringe		Interface motion can be predicted easily	Assumption of a constant value	Disposable on-site diagnostic system	154
5	Food colorant	PDMS	Pipette	0.2–3.7 nl s^−1^	Controlled manner of filling to prevent the collapse	Presence of the resistance	Bioanalytics	155
6	Distilled water	PDMS	Pipette		Controls the speed of the autonomous capillary flow	Experiments were entirely done on distilled water	Total analysis systems or LOCs	156
7		PDMS and PC chips			Longevity is achieved	PC and PDMS chips were made by air plasma treatment		157
8		PDMS with glass slides		1 μl min^−1^	Constant flow rate for more than an hour		LOC devices	158
9	Human serum with CRP	PDMS and glass	Pipette	82 nl min^−1^	Different interface shapes in the PDMS channels	Presence of Laplace pressure stops the flow	Detection of c-reactive protein (CRP)	159
10	Dyed water	PDMS and PMMA			Highly efficient for particle separation	Different channel size is required for different particle filtration	Blood filtration, LOC system	160
11		PDMS	Automated liquid handling systems		Reduce cost and reagent supply			161
12	Blood	PDMS material			Simple to use, flexible and robust	Only about 20 nl of plasma is extracted	POC diagnosis	162
13	Cellulose powder	PDMS and glass surfaces	Pipette		Detection of glucose, protein, and cholesterol	Poor device-to-device reproducibility	Bioanalytical applications or POC diagnostics	163
14	Milk sample	Acrylic resin	Pipette	60 and 110 μl s^−1^	Refill without drying the center channel	Decreased sensitivity	Food analysis and drug discovery	164
15	Bovine serum albumin	PDMS	Pipette		Device to device repeatability is well defined	Pressure prediction at each step is required	Biochemical assays and immunoassays	165
16		PDMS	Due to the rotational speed		Does not require surface treatment		POC diagnosis	183
17	Blood	PDMS mould	Injection moulded		Mostly unidirectional	Error comes from the milling process	Point of care	166
18		PMMA plate	Pipette or micro-needle		Capillary motion of the blood is monitored	Increase in viscosity when velocity decreases	POC diagnosis and home care systems	167
19	Blood	PDMS	Micro-pipette		Increased amount of extracted plasma	Numerous valves are required	POC diagnostics	168
20	Plasma	PDMS	Sweat	7.3 × 10^−3^ to 1.2 × 10^−1^ μl min^−1^	Prolonged flow due to evaporation	Leads to local flow near the inlet	Wearable sweat-sensing device	169
21	Dyed water	PDMS and PMMA	Syringe pump		Separation time is lower	Bend region and the bend angle should be maintained properly	Bioengineering applications and point of care	170
22	Blood	Injection-moulded chip made of COP	Micro-needle		Prevents accidental blood projections induced by forced flow actuation	Leakage can occur based on the substrate materials	Used in haemeagglutination	171
23		PDMS	Syringe pump	1.2 ml min^−1^	Increased stability with a large interface area	Very expensive	Used in the creation of wall-less channels	172
24	Water in oil	PDMS	Solvent delivery systems	3 to 60 μl min^−1^	Flexible manipulation of droplets within the channels	Instability of the droplets that are generated	Drug delivery systems	173
25	Microbeads	PDMS	Syringe-assisted vacuum pumping		Decreased backflow is observed	Non-Newtonian samples do not give a proper assessment	POC or LOC testing applications	174
26		PDMS	Injector	0.865 μl s^−1^	Increase in height increases the self-flowing speed within the channel	Decreased flow due to the rough surface	POC or LOC testing applications	175
27	Red dye in deionized water	PCB fabricated chip	Pipette	138 μl min^−1^	Low-cost, electronic-based disposable analytical platforms		POC diagnosis and LOC equipment	176
28	Acrylamide	Micropads	Syringe pump		Robust in nature		Energy, electronics, medicine, food or cosmetics	177
29	Neuronal cells	3D micropores	Syringe pump	2–2.5 μl min^−1^	Compactable to microscope focusing used in optical tracking	Flow speed cannot be tightly controlled	Brain-on-a-chip	178
30	Mineral oil, silicone oil		Syringe pump	0.005 μl min^−1^	Flexible and reliable, providing possibilities for mono-dispersed droplet-based studies	Presence of viscous and shearing forces	Cell encapsulation and protein crystallization	179
31	Streptavidin-coated fluorescent particles	PVP	Pipette		Three-dimensional cell focusing and on-chip cell sorting	Combines multiple features for analyses	Diagnosing and monitoring clinical conditions	180
32	Neuronal cells	PDMS or glass of choice	Pipette		UV-curable alternative material for low-cost microfluidic chip applications	Tougher analysis of human neural network	Cell capture and cell culture application	181

#### Limitations

Though the capillary circuit and capillary elements contribute many characteristics to microfluidics, they are unable to deliver self-regulated flows for performing advanced functions.^184^ The capillary action depends on the surface tension and adhesion of the analyte to the channel surfaces.^151^ The concentration of fluid in the outlet reservoir limits the overall flow duration; however, to alleviate this, multiple outlet reservoirs are used to reduce the rate of concentration in each reservoir to maintain longer flow duration.^158^

### Gravity-driven flow

**Fig. 8:**
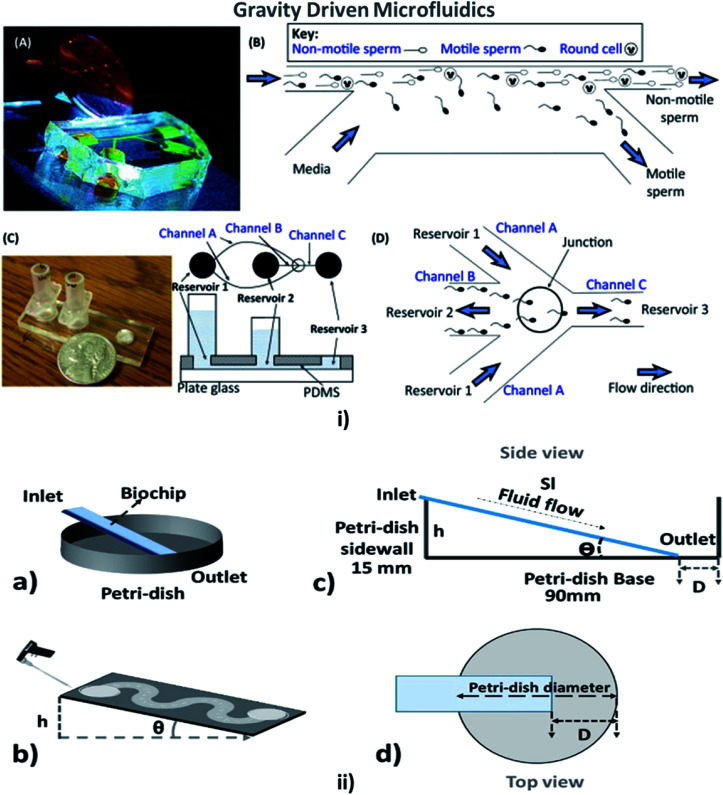
(i) (A) A compact, simple, and disposable device for passively driven sperm sorting; (B) fluid flow used to sort sperm based on their ability to cross the passively driven laminar fluid stream created by a hydrostatic pressure difference between the inlet and outlet (this figure has been adapted from ref. 186 with permission from ACS Publications, copyright: 2003). (C) A compact, passively driven microfluidic device with a side-view and top-view schematic diagram of the generation of hydrostatic pressure differences (this figure has been adapted from ref. 198 with permission from Springer, copyright: 2007). (D) The junction showing sperm movement from an input (reservoir 2) to an outlet (reservoir 3) (this figure has been reproduced from ref. 199 with permission from ELSEVIER, copyright: 2015). (ii) (a, b) Illustrations of pipette Petri dish single-cell trapping (PP-SCT) or tilt microfluidic trapping; (c, d) the side and top view, respectively, where *θ* is the tilt angle, *h* is the height of the inlet, *D* is the distance between the biochip outlet side edge to the Petri dish wall along the diameter, and Sl is the glass slide length (this figure has been adapted from ref. 196 with permission from MDPI, copyright: 2018).

This is a technique in which the fluid flow is driven or assisted by the earth's gravity, depending on the viscosity of the fluid and the height of the inlet from the surface. A comfortable and straightforward analysis of the flow rate in the MFD can be determined with the help of the gravity-driven flow principles which accelerate the passive pumping system was reported by Mäki *et al.* in 2014.^185^ The microfluidics and LOC devices utilizing the gravity for their passive operations are shown in Fig. 8.

In 2003, Cho *et al.* described a simple disposable polymeric microscale integrated sperm sorter (MISS) to isolate sperm from semen, which was unattainable by traditional sperm-sorting methods.^186^ Similarly, Suh *et al.* explained the purpose of obtaining the motile sperm through gravity-driven flow with increased morphology from normal semen.^187^ Not only sperm but blood cells could be counted using a gravity-driven MFD that was fabricated using UV laser ablation and resin film lamination by Yamada *et al.* In 2005.^188^ Huh *et al.* developed a unique combination of a simple, self-contained microfabricated device using field-driven separation with micro-hydrodynamics-assisted separation, where the earth's gravity was used to drive the fluids across the channel to assist in separation.^189^ For cell culture, Lee *et al.* designed a compatible passive gravity-driven MFD with long-term continuous cell culture perfusion, which was adapted from the standard 96-well plate format to remove tubing and connectors.^190^

In 2010, Zhang *et al.* presented a gravity-actuated technique where an oil phase was loaded into the infusion set and then the water phase was pumped into the PTFE (polytetrafluoroethylene) tubing by the gravity of the oil phase.^191^ Thereby, Sung *et al.* developed a gravity-induced-flow MFD to provide long-term flow by eliminating the bubble formation based on the mathematical PK-PD (pharmacokinetic/pharmacodynamic) model.^192^ Abaci *et al.* in 2014 developed a pumpless recirculating gravity-driven human skin equivalent (HSE)-on-a-chip platform that was simple to fabricate, handle and operate when placed on a rocking platform.^22^ A way of inducing fluid flow, by elevating the inlet of the channel against gravity by placing it on a rocking platform was demonstrated by Esch *et al.* in 2015.^193^ A compactly configured differential flow resistance microfluidic single-cell trapping device with a shorter flow path was introduced and demonstrated by Jin *et al.* to increase the speed and throughput (in both mathematical and numerical simulations).^194^ James *et al.* developed an MFD with efficient trapping of single cells through hydrodynamic flow by positioning the microwells along the flow path, which acted as a mechanical barrier. The hydrodynamic flow inside the channel was analyzed with Comsol Multiphysics with different boundary conditions for varying particle sizes.^195^ Simultaneously, Narayanamurthy *et al.* described the rate of single-cell trapping based on the shape of the channel in a passive biochip and concluded that hexagonally positioned microwells possessed high single-cell capture (SCC) percentages. The SCC potential of microfluidic biochips was found to be an improvement over straight channels, branched channels, or serpentine channels. Multiple cell capture (MCC) began to decrease from the straight channel, branched channel, or serpentine channels.^196^ Kim *et al.* presented a design for gravity-driven microfluidic systems that could generate self-switching pulsatile flows to mimic physiological blood flow pulsing.^197^ Comprehensive developments in gravity-based passive pumping in microfluidics are summarised in Table 5.

**Table 5:** Recent developments in gravity-based passive pumping in microfluidics

S. no	Analytes used	Materials used	Auxiliaries involved	Flow rate	Advantages	Disadvantages	Applications	Ref. no
1	Semen	PDMS		0.008 μl s^−1^	Readily fabricated device	Very low flow rate	Vitro fertilization procedures	186
2	Semen	PDMS, glass	Pipette	∼20 ± 40 μl h^−1^	Inexpensive, portable, easy to use, and disposable	Does not isolate every motile sperm effectively	Chemical and biological analysis, immunoassay	187
3	Blood, physiological salt solution	Silicon laminated with resin	Pipette, syringe		One-stroke fabrication of grooves for channels	Combining the laminar flows at the microchannel is difficult	Biology and molecular genetics	188
4	Fluorescein dissolved in BSA solution	PDMS	Tubing	1 ml h^−1^	Continuous particle separation is achieved	Increasing the flow rate will lead to low purity separation for a larger drop	Ultrasound imaging	189
5	DMEM with no drug	PDMS, glass	Pipette	50 μl per day	Multiple culture plates can be incubated	Requires specialized equipment for cell loading	Cell-based screening, cytotoxicity assay	190
6	Blood and lysing buffer	PMMA			Efficient lysis of RBC with simple structure	Cellular debris cleaning should be done constantly	Clinical genetics, diagnostics	200
7	Dimethyl silicone oil	PDMS	Turntable and infusion set		Droplet generation, transport, collision, fusion, mixing, and stopping are possible	Hard to generate droplets for a long period	Cell research and high-content drug screening	191
8	Cell culture medium	Multilayers of PDMS			Prevents formation of air bubbles, up to 3 days of operation	Nutrient depletion and accumulation of waste affects the viability of cells	Prediction of drug toxicity, cell culture	192
9	DMEM with 10% FBS	PDMS membrane	Injection		Long-term maintenance of HSEs for drug testing purposes	Unstable flow rate with decreasing reservoir volume	Skin drug testing studies	22
10				650 μl min^−1^	Evaluate drugs under fluidic cell culture conditions			193
11	Human cervical carcinoma and embryonic kidney cells	PDMS	Pipette	375 μl min^−1^	Highly beneficial as the rare cells are trapped	Non-uniform distributions of fluidic velocities and pressure drops	Signalling pathway activation, and inhibition (in SCA)	194
12	Water				Increased particle trapping on using a serpentine channel		Single-cell trapping based on the interest	195
13	Motile and non-motile sperm, water	PDMS, glass	Syringe		Highly compatible	Flow is dependent on the difference in height of the source reservoir	Cell culture	197
14	Trypan blue and lung cancer cells (A549)	Glass substrate	Mechanical obstacles	0.25–4 ml s^−1^	Highly efficient single-cell trapping	Area density of single-cell arrays are reduced	Genomics, proteomics, secretomics	196

#### Limitations

For specific cell culture applications, dynamic or pulsatile flow was necessary, while gravity-driven microfluidic systems could only generate continuous flow.^57^ However, with the insertion of a periodic rocking device, an active pump would be suitable to produce a dynamic flow.^192^ Upon tilting, this method for establishing an air–epidermal interface within a gravity-driven flow system is highly complex. Hence, the reservoir volume was subjected to minor adjustments and properly levelled surface for tilting.^22^

### Hydrostatic pressure

Hydrostatic pressure is the force exerted by a fluid at equilibrium at a given point acting with equal magnitude in all directions, due to the force of gravity. Hydrostatic pressure increases in proportion to the depth measured from the surface because of the increasing weight of the fluid, exerting a downward force from above.^201^

**Fig. 9:**
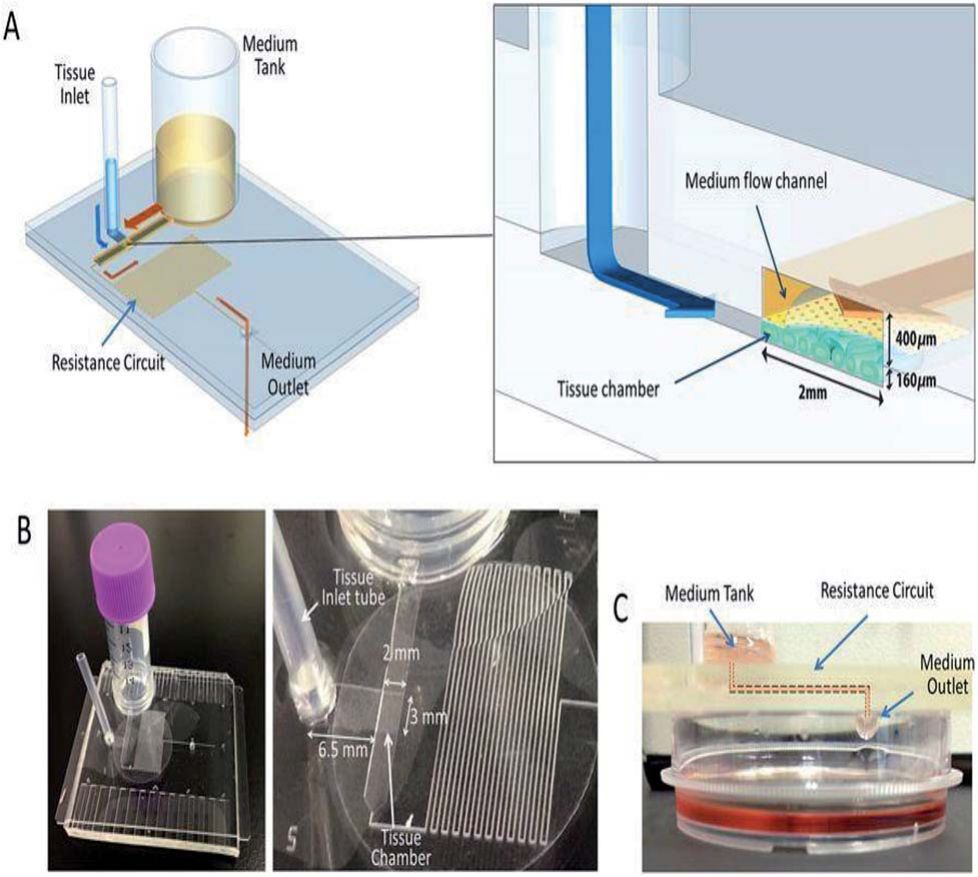
A pumpless MFD. (A) A schematic 3-D image of the PL MFD, showing the sample tank, tissue inlet, resistance circuit, and sample outlet, and an enlarged view of the portion encompassing the tissue chamber. (B) Pictures of the device. On the right, a closer view of the tissue chamber and resistance circuit. (C) A low-lateral view of the device demonstrating the medium-flow route, finally dropping down to the collecting dish (this figure has been reproduced from ref. 205 with permission from Springer, copyright: 2017).

Weigl *et al.* presented hydrostatic pressure-driven microfluidic elements, including mixers, valves, and detectors that were employed in ultra-low-cost disposable qualitative and semi-quantitative medical and environmental assays for the home, office, and field use, and for sample or reagent preparation tools to provide processed liquids for downstream analysis.^202^ Subsequently, a microfluidic cartridge was planned for the extraction of small molecules by the hydrostatic pressure from the mixture of small and large molecules.^203^ For the culture medium, Marimuthu *et al.* developed a pumpless perfusion microfluidic chip that could deliver a constant flow rate with reduced pressure due to the intravenous (IV) setup used at the inlet, over the siphon-based gravity-driven microfluidics.^204^ Later, Seo *et al.* employed hydrostatic pressure to sort motile sperms of three species, namely bull, mouse, and human, with an average sorting rate.^198^ The culturing of mouse testis tissue and spermatogenesis in a hydrostatic pressure environment were developed by Komeya *et al.* In addition, researchers used a resistance circuit to induce slow and long-lasting medium flow in the channel.^205^ MFD and LOC devices employing hydrostatic pressure for their passive operations are shown in Fig. 9. Comprehensive reports on the hydrostatic pressure-driven passive pumping technique in microfluidics are summarised in Table 6.

**Table 6:** Recent works on hydrostatic pressure-driven passive pumping technique in microfluidics

S. no	Analytes used	Materials used	Auxiliaries involved	Flow rate	Advantages	Disadvantages	Applications	Ref. no
1	Indicator dye with blood		Micropipette or syringe	99 nl s^−1^	Low cost, portable	Equilibrium constant affects the flow rate	Blood plasma separations, blood typing, qualitative immunoassays	202
2	DNA sample, blood	Plastic				Prediction for high-viscosity solutions is difficult	Drug discovery, toxicology	203
3	Human dermal fibroblast neonatal cells		Silicon tubing and syringe	0.1–10 ml min^−1^	Constant flow rate is achieved	Can control only a slow perfusion rate and not suitable for all cells	Development of artificial skin and long-term cell-culture	204
4	Sperm with phosphate buffered solution	PDMS	Self-movement		Alignment and orientation of the sperm is possible	Height change in the reservoirs disturbs the fluid flow	Clinical labs	198
5	Mouse testis tissue	PDMS	Micropipette	0.05 μl min^−1^	Constant flow rate for a long range is observed		Tissue culture and organ culture	205

#### Limitations

In a few cell cultures and trapping experiments, a more accurate flow rate was not obtained due to the reservoir design of the device. This could be modified with suitable adjustments in the diameter of the reservoir with the constant supply of fluids to the inlet.^205^

### Vacuum driven

A vacuum refers to any space in which the pressure is lower than atmospheric pressure (negative pressure). Vacuum-driven devices use the ability of an MFD to suck the sample through negative pressure without any extra on/off-chip microfluidic units. Low vapour pressure and degassing become essential when the vacuum pressure falls below this vapour pressure. Degassing is the process of removal of any gas in the channel through permeability or solubility within the membrane to generate a vacuum.^206^ Song *et al.* worked to produce a better demonstration of vacuum degassed flow in POC applications using a PDMS-based material, coated with Parylene C.^207^

Later, Monahan *et al.* developed a channel outgassing technique, where the channel was evacuated, and the negative pressure generated inside the channel assisted the flow with 90% efficiency in eliminating bubble formation.^208^ A new power-free pumping method for PDMS MFD was developed by Hosokawa *et al.* to overcome a significant issue in the detection of gold nanoparticle DNA analysis.^75^ Subsequently, they presented an on-chip heterogeneous immunoassay with a simple structure and operational procedure. Redissolution through the microchannel walls developed the capacity to drive the solution movement and prevent air bubble trapping.^209^ Dimov *et al.* reported a microfluidic blood analysis system where the plasma was separated by trapping the RBC and WBC in a trench, and the inlet fluid was pumped due to the suction of the chamber, as a result of pre-evacuating the channel.^210^ Liang *et al.* developed a degassed channel with negative pressure to pump the fluid irrespective of the surface tension of the fluid. Before sample loading, channel geometry, surface area, PDMS thickness, exposure area, vacuum degassing time, and post-vacuum idle time when the device was exposed to atmospheric conditions were inspected.^211^ Eventually, Li *et al.* designed a PDMS modular pump in a vacuum desiccator with high flexibility and reduced fabrication complexity to create negative pressure in the channel for sample movement.^212^

Furthermore, Li *et al.* developed a self-powered one-touch finger-press-activated blood extraction system based on the negative pressure-driven force developed in the pre-vacuum actuator.^213^ Xu *et al.* designed a vacuum-driven power-free MFD that depended on the gas solubility or permeability of PDMS that restored the air inside it and encouraged the transfer of air into a vacuum.^214^ Also, Li *et al.* worked on the development of a self-powered PDMS-based microfluidic droplet generator with mono-dispersed droplet generation and multi-sample introduction in a controlled way.^215^ A simplified and inexpensive passive microfluidic channel with excellent analytical performance to carry out microflow injection analysis (mFIA) was investigated by Agustini *et al.*^216^ Subsequently, Liu *et al.* developed a paraffin wax and glass MFD through higher driving pressure to maintain longer working times where the inlet was exposed to air with a slower decline in flow rate.^217^ Li *et al.* proposed a high-pressure MFD to drive the fluid using gas permeation and suppressing the generation of bubbles under high temperatures.^218^ Then, a power-free and self-contained fluid reactor array that performed parallel fluid loading, metering and mixing through pre-degassed PDMS and the change in capillary force due to sudden narrowing of the channel cross-section was developed by Liu *et al.*^219^

#### Diffusion

This is the movement of solute particles from a region of higher concentration to a region of lower concentration due to the random motion of atoms or molecules with or without a semi-permeable membrane. Warrick *et al.* compared the theoretical and experimental data of microchannels and standard wells on the metrics of sample washing and experimental error in treatment concentrations.^220^

#### Permeation

**Fig. 10:**
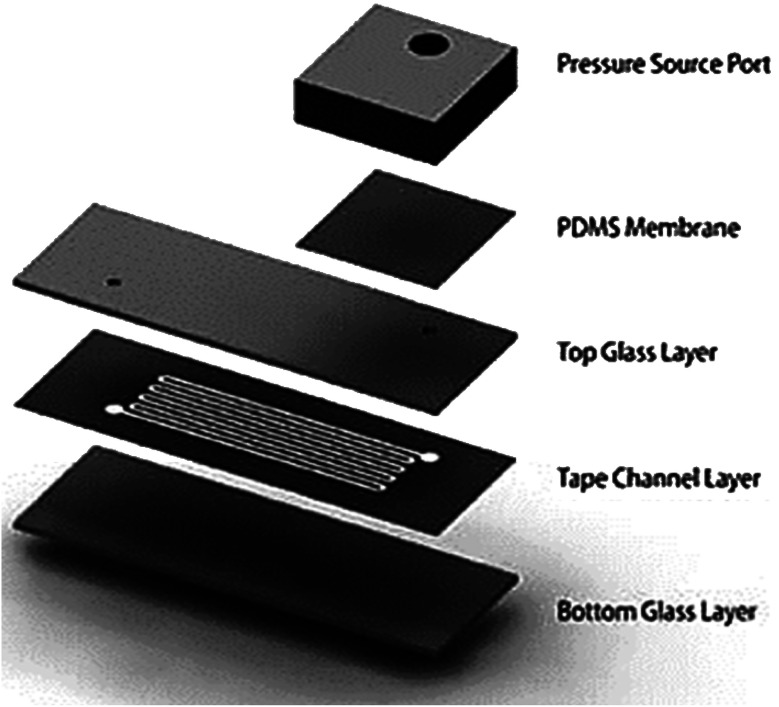
A hybrid PDMS utilizing permeability and capillary effects for fluid flow (this figure has been reproduced from ref. 225 with permission from IOP, copyright: 2009).

**Fig. 11:**
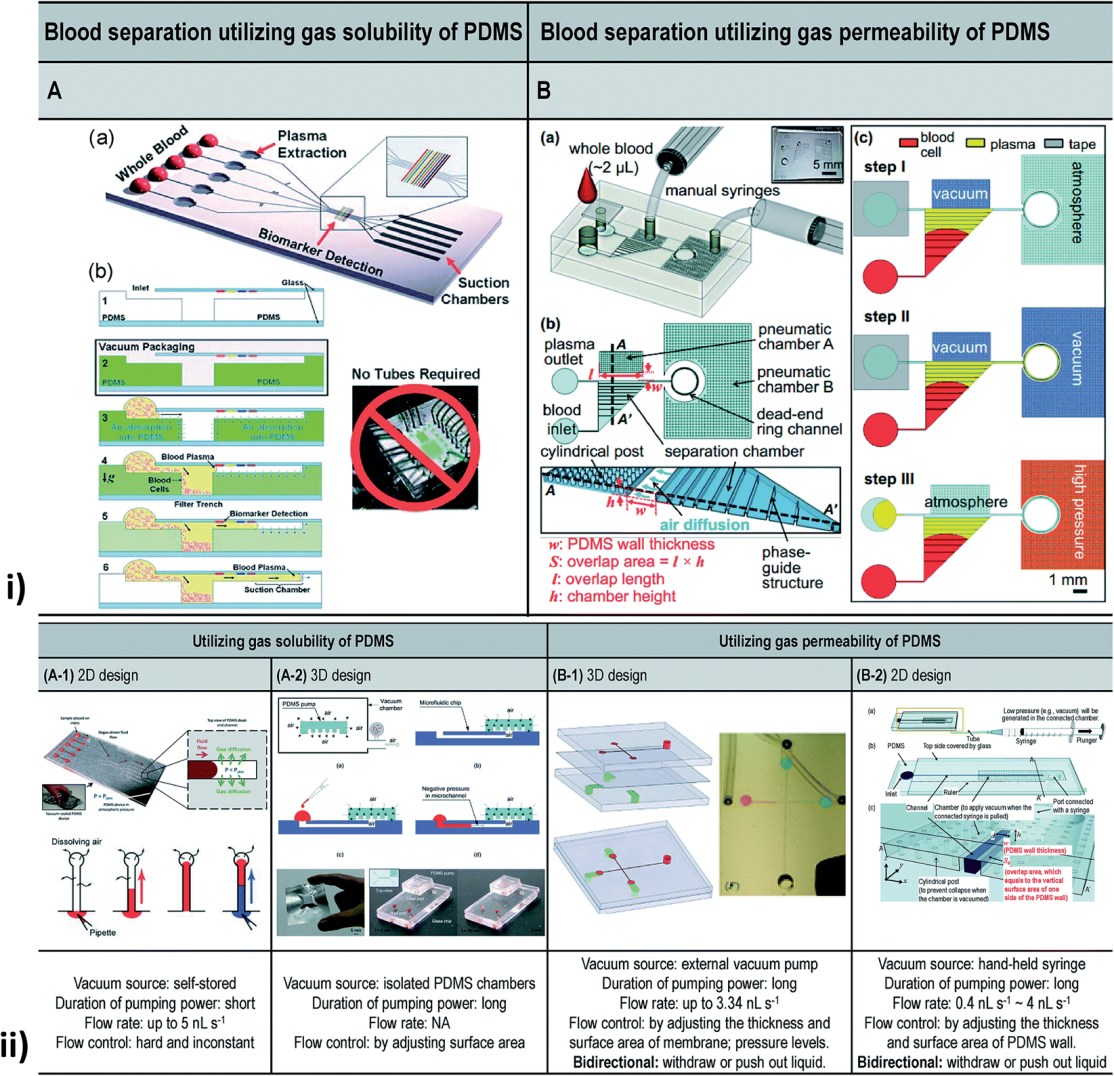
(i) Blood separation utilizing gas solubility and the permeability of PDMS. [A] Self-priming, self-contained, tether-free SIMBAS. [B] Schematic diagrams of the proposed device. (B-a) An overview of the experimental setup using the proposed device. The top layer is a PDMS cover with an inlet and a tape-sealed outlet, and is bonded irreversibly with a bottom fluidic layer. (B-b) A top view and cross-section view. The separation chamber is divided into ten segments of equal volumes by nine phase guides at the bottom. Cylindrical posts are used to prevent the collapse of the pneumatic chamber when it is evacuated by the manual syringes. w and S stand for the PDMS wall thickness and the overlap area between the pneumatic chamber A and the separation chamber, respectively. The overlap area (S), where the flux of air diffuses, is calculated from the overlap length (l) multiplied by the chamber height (h). Drawings are not to scale. (B-c) Experimental steps. (ii) Different types of vacuum-driven power-free micro-pumping methods utilizing the gas solubility or permeability of PDMS. (A-1) A 2D micro-pumping design utilizing the gas solubility of PDMS. A whole PDMS device is pre-evacuated in a vacuum environment. (A-2) A 3D micro-pumping design utilizing the gas solubility of PDMS. A PDMS slab is pre-evacuated in a vacuum environment. See also Fig. 1A. (B-1) A 3D micro-pumping design utilizing the gas permeability of PDMS. External vacuum pumps are connected to the ports in the control channels. (B-2) A 2D micro-pumping design utilizing the gas permeability of PDMS. A hand-held syringe can generate a vacuum environment (figures (i) and (ii) have been adapted from ref. 214 with permission from the Royal Society of Chemistry, copyright: 2015).

Verneuil *et al.* described the process of water permeation in microstructures through silicone which has a strong influence on fluid displacement.^221^ Randall *et al.* confirmed the use of lubrication approximation to solve gap-averaged velocity while transferring water from a single rectangular channel into a PDMS using permeation-driven flows.^222^ Eddings *et al.* confirmed that the fluid flow in a PDMS-based MFD was generated through high gas permeability at the membrane.^223^ The evaporation of water in the polymer walls through permeation resulted in fluid flow due to the excess pressure stored in the compartment.^224^ A hybrid PDMS utilizing permeability for fluid flow is shown in Fig. 10. MFD and LOC devices using a vacuum for their passive operation are shown in Fig. 11. Comprehensive developments in vacuum/permeation-driven passive pumping in microfluidics are summarised in Table 7.

**Table 7:** Recent works on vacuum/permeation driven passive pumping in microfluidics

Sl. no	Analytes used	Materials used	Auxiliaries involved	Flow rate	Mechanisms used	Advantages	Disadvantages	Applications	Ref. no
1	Aqueous fluorescent buffers spiked with fluorescein	PDMS	Syringe		Degassed driven flow	Efficient at eliminating air bubbles	Channel networks are complex in construction		208
2	Fluorescent particle solution diluted with deionized water	PDMS	Micropipette	0.5–2 nl s^−1^	Degassed driven flow	Efficient deposition and two analyses can be conducted on both sides	Not suitable for continuous operations	Point-of-care, single-use analytical devices	75
3	Human serum enriched with CRP	PDMS with SU-8 photoresist and glass	Pipette	3–5 nl s^−1^	Degassed driven flow	Fast assay time is achieved	Usage of deionized water for dilution	Solution mixing and electrophoresis	209
4	Blood	PDMS		0.5–2 nl min^−1^	Degassed driven flow	Blood analysis is computed within 10 minutes	Evacuation of the channel is necessary	POC diagnosis	210
5	Blue food colouring dye	PDMS placed on glass slides	Micropipette	0.2 to 3 nl s^−1^	Degassed driven flow		Degassing time is very high	Lab-on-a-chip devices	211
6	Food dye solution	PDMS	PDMS pump slab, micropipette		Degassed driven flow		Pump slab must be evacuated overnight	LOC devices and point-of-care diagnostic	212
7	Blood (rabbit)	PDMS	Micro-needle		Degassed driven flow			Point-of-care diagnosis	213
8	Bovine serum	PDMS	Sequential injection		Degassed driven flow	No need for surface treatment	Pumping power decreases at a faster rate	Point-of-care diagnosis	214
9			Plastic pipette tips		Degassed driven flow	Self-powered and portable for easy convenience	Fluctuation of the flow rate is seen	Droplet-based applications in in-field analysis	215
10		Cotton threads	Injection moulded	2.2 ± 0.1 μl s^−1^	Degassed driven flow	Simple, fast, cheap and reliable in their application	Loss of samples remains unavoidable		216
11	Food dye solution	PDMS, glass substrate		0.92 μl min^−1^	Degassed driven flow	Longer pumping duration towards the outlet	Immediate decay is possible due to diffusion	Applications with longer working period	217
12	Aqueous/oil microdroplet	PDMS	Pipette	250 μl h^−1^	Degassed driven flow	Dramatic improvement of pumping performance	Bubble generation is observed under vacuum desiccators	Autonomous microdroplet-generation/transport and biometrics	218
13	Dye	PDMS	Pipette		Degassed driven flow	Inexpensive in their construction and implementation	Highly expensive at their maintenance and insufficient instrumentation	Assay, protein crystallization, drug discovery, and combinatorial chemistry	219
14	Water droplet	PDMS on glass	Injector		Permeation driven flow	Adhesion is achieved through low pressure	Mixture of liquids cannot be driven	Concentrate colloids and crystallize those particles	221
15	Water droplet	PDMS with two fluid reservoirs	Pipette		Permeation driven flow	Formation of layered microstructures at the center due to permeability		Bead stacking, chemical concentration, and passive pumping	222
16	Red, blue, and green coloured fluids	PDMS	Syringe	200 nl min^−1^	Permeation driven flow	Bubble-free flow throughout the membrane	Complete filling of the channels is required	Drug delivery and micro total analysis	223
17	Dimethyl sulfoxide (DMSO)	PDMS with autopsy needle	Syringe		Permeation driven flow	Multiple fluids can be integrated		Bioanalytical application	224
18	Plasma of blood	PDMS and glass slide	Syringe		Diffusion	Improved accuracy and precision	Evaporation effects were witnessed	Drug screening	220

#### Limitations

Due to the limited air diffusion and hydrophobicity of the PDMS material, the flow rates acquired were reduced to nanoliters per second. This could be enhanced by increasing the surface area of the material used.^223^

## Critical discussions and limitations

The evolution of new microfluidic tools for genomics, proteomics, and metabolomics is progressing swiftly in research laboratories and will provide the motive for large-scale production. Passive flow methodologies, such as surface-tension-driven flow, capillary-based flow, gravity-driven flow, hydrostatic-pressure-driven flow, and osmosis-driven flow techniques are found to be suitable in assisting the flow without any external sources. Despite the beneficial properties of PDMS that permitted its fast enactment in applied fields, there are several limitations in using the material in biomedical research.^226^ The control of fluid flow over space and time with sufficient accuracy was the fundamental challenge in designing MFDs.^227^ The initial fabrication of MFDs required clean-room facilities for silicon or glass devices which were eliminated by polymers and elastomers devices. However, PDMS was limited by its ability to withstand high temperature, difficulty in developing complex multi-layered 3D-configured devices, and incompatibility with organic solvents and low molecular weight organic solutes due to their surface chemistry.^228^ The usage of inert chemicals and utilization of high-throughput methods for manufacturing and permeability limit the application of PDMS devices in a few cases.^229^ Evaporation affects the application of microfluidics in various domains.^230^ This could be overridden by maintaining a uniform surface throughout the entire channel area. In pressure-driven techniques, variation in pressure is noted due to the difference in channel length and utilization of different fingers at the inlet.^119,127^ This unbalanced pressure may lead to backflow, followed by clogging or the entire destruction of the device. Hence, a uniform pressure needs to be maintained for a long period. This can be achieved by osmosis-driven MFDs. However, in osmosis-driven MFDs, the osmotic reagent had to be refreshed at regular intervals, which restricted their applications.^145^ In addition to that, selection of a suitable membrane is necessary to maintain the pH and to avoid clogging when biological cells or biomolecules are used.^143^ Hence, capillary-based passive flow techniques are used as they depend only on the design and material characteristics of the MFD. On the other hand, in some instances, due to a drop in flow rate, essential pressure prediction at each step decreased sensitivity in capillary-based passive flow techniques.^151,165^ This could be averted by selection of suitable materials with the ability of spontaneous sample filling in the column.^231^ Because of this, under many circumstances, gravity-driven techniques are adopted, but they generate an unstable or decreasing flow rate, which was further eliminated by maintaining an appropriate reservoir volume and proper inlet pressure.^22,198^ Whereas hydrostatic-pressure-driven techniques are limited by the development of Laplace pressure at the air–liquid interface due to the affinity between the liquid, the atmosphere, and the reservoir parameters.^232^ Another listed shortcoming was the linear pressure drop with time. These errors could be avoided, if the pressure difference was obtained by varying the altitude of the liquid to the atmospheric interface in the proper way.^197^ They could be eliminated by using a vacuum MFD; nevertheless evacuation is necessary to drive the fluid in order to reduce the pressure within the channel. Due to the presence of high pressure initially, the suction of the fluid is higher and gradually decreases as the pressure decreases. Hence a syringe pump is used to maintain a constant flow in the device. This additional requirement can be neglected by the use of simple capillary force.^211^ Based on the study performed, the vacuum-driven techniques with complex construction were not suitable for continuous operation due to the evaporation effect and the loss of sample in a few cases.^75,208,216^ Besides the above-mentioned limitations, these methods found numerous clinical applications, because they use ultra-low volumes of biofluids for processing and can be accomplished quickly and efficiently.

### Industrial perspectives

One of the promising types of assistance of microfluidics is found in fast, reliable, and accurate POC diagnostic devices. Detection and diagnostics of communicable diseases can decrease the mortality rate in many developing and less developed countries. As reported by the World Health Organization (WHO), nearly 46% of new tuberculosis case are not detected, causing over 3 million incidences to be missed annually.^233,234^ Also, according to a recent report by WHO, the lack of proper diagnostic capacity is a significant challenge in monitoring effective service coverage. As an example, cholera was estimated to have infected 1.3–4 million people annually and to have caused 21 000–143 000 deaths each year during 2008–2012. But the average annual numbers of cases and deaths reported to WHO were only 313 000 and 5700, respectively. One of the major issues is the lack of diagnostic capacity, which results in the exact burden of the disease being unknown, thus making it difficult to take preventive measures.^235^ Although many researchers in the microfluidic domain are focusing on developing diagnosis and detection devices – searching for a few related keywords such as diagnosis, detection, and pathogen detection in LOC, bio-microfluidics, and biomedical microdevices resulted in nearly 3000 papers – many of the products do not end up on the market. One major issue in this regard is transforming common expensive sample delivery systems in laboratories, such as syringe pumps, into passive and low-cost delivery systems to meet the needs of the market in developing and less developed countries. To analyse the status of current commercialized MFDs, we delved into the circle database of uFluidix Inc (http://circle.ufluidix.com). The database includes a list of registered start-ups offering microfluidic products. Nearly 85% of the analysed start-ups in the diagnostics industry were utilizing a passive method for sample delivery. This clearly emphasizes the importance of passive techniques for industrial sectors.

## Conclusion and future directions

Microfluidics is articulated as a multidisciplinary research field that requires basic knowledge in fluidics, micromachining, electromagnetics, materials, and chemistry to find their relevance in the pharmaceutical industry, diagnosis, healthcare, and life science research. LOC is one of the essential applications of microfluidics, and is also a revolutionary tool for many varieties of applications in chemical and biological analyses due to its fascinating advantages (speed, low cost, simplicity, and self-testing) over conventional chemical or laboratory equipment.

Microfluidics covers the science of fluidic behaviours at micro/nanoscales employed in design engineering, simulation, and fabrication of fluidic devices. It is the backbone of biological or biomedical microelectromechanical systems (BioMEMS) and the LOC concept, as most biological analysis involves fluid transport and reaction. MFDs have been operated in inkjet printing, blood analysis, biochemical detection, chemical synthesis, drug screening/delivery, protein analysis, DNA sequencing, and so on. Several different MFDs have been developed with basic structures analogous to macroscale fluidic devices. Such devices include microfluidic valves, microfluidic pumps, and microfluidic mixers. Active devices are usually more expensive, due to their functional and fabrication complexities. However, it has been very challenging to implement these actuation schemes fully at the microscale, owing to the requirements for high voltages, electromagnets, *etc.* Typically, passive MFDs do not require an external power source, where control is instituted by the energy drawn from the working fluid, or based purely on surface effects, such as surface tension or fluid pressure, with high reliability due to the lack of mechanical wear and tear. Hence, upcoming industrial sectors rely on passive methods to achieve better results in a composed way.

MFDs offer very repeatable performance, once the underlying phenomena are well understood and characterized. They are well suited to bioMEMS applications, as they can handle several microfluidic manipulation sequences. They are also well suited to the low-cost mass production of disposable MFDs to specifically work with blood. In addition, inexpensive and simple fabrication techniques can promote the use of paper-based MFD in various research fields, such as drug testing and viral detection.

Passive MFDs with high throughput, high flow rate, reduced operation time and easy equipment handling are expected to have profitable outcomes. Therefore, MFDs with passive methods are designed in such a way as to offer a wide range of optimizations that can be performed within the channel and reservoir dimensions for employment to certain specific applications with better flow rates. In the future, device progress will focus on developing new materials for substrates in such a way as to overcome the drawbacks of currently existing devices. Passive systems cannot maintain constant flow rates. Hence, systems with constant flow rates are expected to be developed as an advantageous method. In addition, a low operation time remains an essential parameter. A reduction in running time can be demonstrated to be the crucial factor on which to focus during upcoming developments. Future work is expected to focus on exploring these listed areas, so that microfluidics can find application in many other fields to satisfy growing demands and needs.

## Conflicts of interest

There are no conflicts of interest to declare.

## Supplementary Material

## References

[cit1] Lauga E. , Brenner M. and Stone H., in Handbook of Experimental Fluid Mechanics, Springer, Heidelberg, 2007, ch. 19, pp. 1219–1240, 10.1007/978-3-540-30299-5_19

[cit2] Stone H. A., Stroock A. D., Ajdari A. (2004). Annu. Rev. Fluid Mech..

[cit3] Whitesides G. M. (2006). Nature.

[cit4] Nge P. N., Rogers C. I., Woolley A. T. (2013). Chem. Rev..

[cit5] Zhu L., Jiang G., Wang S., Wang C., Li Q., Yu H., Zhou Y., Zhao B., Huang H., Xing W., Mitchelson K., Cheng J., Zhao Y., Guo Y. (2010). J. Clin. Microbiol..

[cit6] Singh P. (2017). J. Comput. Appl..

[cit7] Volpatti L. R., Yetisen A. K. (2014). Trends Biotechnol..

[cit8] Chokkalingam V., Tel J., Wimmers F., Liu X., Semenov S., Thiele J., Figdor C. G., Huck W. T. (2013). Lab Chip.

[cit9] Jain K. K. (2000). Pharmacogenomics.

[cit10] Ghallab Y., Badawy W. (2004). IEEE Circ. Syst. Mag..

[cit11] Saggiomo V., Velders A. H. (2015). Adv. Sci..

[cit12] Dittrich P. S., Manz A. (2006). Nat. Rev. Drug Discovery.

[cit13] Sia S. K., Kricka L. J. (2008). Lab Chip.

[cit14] Kost G. J., Tran N. K., Tuntideelert M., Kulrattanamaneeporn S., Peungposop N. (2006). Am. J. Clin. Pathol..

[cit15] Wu M. H., Huang S. B., Lee G. B. (2010). Lab Chip.

[cit16] Wu L. Y., Di Carlo D., Lee L. P. (2008). Biomed. Microdevices.

[cit17] Kang L., Chung B. G., Langer R., Khademhosseini A. (2008). Drug Discovery Today.

[cit18] Kleinstreuer C., Li J., Koo J. (2008). Int. J. Heat Mass Transfer.

[cit19] Liu Z.-B., Zhang Y., Yu J.-J., Mak A. F.-T., Li Y., Yang M. (2010). Sens. Actuators, B.

[cit20] Toh Y. C., Lim T. C., Tai D., Xiao G., van Noort D., Yu H. (2009). Lab Chip.

[cit21] Yu L., Chen M. C., Cheung K. C. (2010). Lab Chip.

[cit22] Abaci H. E., Gledhill K., Guo Z., Christiano A. M., Shuler M. L. (2015). Lab Chip.

[cit23] Khetani S. R., Berger D. R., Ballinger K. R., Davidson M. D., Lin C., Ware B. R. (2015). J. Lab. Autom..

[cit24] Martel J. M., Toner M. (2013). Sci. Rep..

[cit25] Kr$uuml$ger J., Singh K., O$apos$Neill A., Jackson C., Morrison A., O$apos$Brien P. (2002). J. Micromech. Microeng..

[cit26] Dittrich P. S., Schwille P. (2003). Anal. Chem..

[cit27] Yang S.-Y., Hsiung S.-K., Hung Y.-C., Chang C.-M., Liao T.-L., Lee G.-B. (2006). Meas. Sci. Technol..

[cit28] Baret J. C., Miller O. J., Taly V., Ryckelynck M., El-Harrak A., Frenz L., Rick C., Samuels M. L., Hutchison J. B., Agresti J. J., Link D. R., Weitz D. A., Griffiths A. D. (2009). Lab Chip.

[cit29] Bhagat A. A., Bow H., Hou H. W., Tan S. J., Han J., Lim C. T. (2010). Med. Biol. Eng. Comput..

[cit30] Shields C. W. t., Reyes C. D., Lopez G. P. (2015). Lab Chip.

[cit31] Autebert J., Coudert B., Bidard F. C., Pierga J. Y., Descroix S., Malaquin L., Viovy J. L. (2012). Methods.

[cit32] Zhu X., Yi Chu L., Chueh B. H., Shen M., Hazarika B., Phadke N., Takayama S. (2004). Analyst.

[cit33] Gu W. , Zhu X., Futai N. and Cho B. S. and Takayama S., presented in part at the 7th lnternational Conference on Miniaturized Chemical and Blochemlcal Analysts Systems, California USA, 2004

[cit34] Hung P. J., Lee P. J., Sabounchi P., Lin R., Lee L. P. (2005). Biotechnol. Bioeng..

[cit35] Peterson S. L., McDonald A., Gourley P. L., Sasaki D. Y. (2005). J. Biomed. Mater. Res., Part A.

[cit36] Lee P., Lin R., Moon J., Lee L. P. (2006). Biomed. Microdevices.

[cit37] Gomez-Sjoberg R., Leyrat A. A., Pirone D. M., Chen C. S., Quake S. R. (2007). Anal. Chem..

[cit38] Ong S. M., Zhang C., Toh Y. C., Kim S. H., Foo H. L., Tan C. H., van Noort D., Park S., Yu H. (2008). Biomaterials.

[cit39] Lecault V., Vaninsberghe M., Sekulovic S., Knapp D. J., Wohrer S., Bowden W., Viel F., McLaughlin T., Jarandehei A., Miller M., Falconnet D., White A. K., Kent D. G., Copley M. R., Taghipour F., Eaves C. J., Humphries R. K., Piret J. M., Hansen C. L. (2011). Nat. Methods.

[cit40] Mehling M., Tay S. (2014). Curr. Opin. Biotechnol..

[cit41] Sista R., Hua Z., Thwar P., Sudarsan A., Srinivasan V., Eckhardt A., Pollack M., Pamula V. (2008). Lab Chip.

[cit42] Myers F. B., Lee L. P. (2008). Lab Chip.

[cit43] Sackmann E. K., Fulton A. L., Beebe D. J. (2014). Nature.

[cit44] Weibel D. B., Whitesides G. M. (2006). Curr. Opin. Chem. Biol..

[cit45] Gravesen P., Branebjerg J., Jensen O. S. (1993). J. Micromech. Microeng..

[cit46] Stone H. A., Kim S. (2001). AIChE J..

[cit47] McDonald J. C., Duffy D. C., Anderson J. R., Chiu D. T., Wu H., Schueller O. J. A., Whitesides G. M. (2000). Electrophoresis.

[cit48] Fujii T. (2002). Microelectron. Eng..

[cit49] McDonald J. C., Whitesides G. M. (2002). Acc. Chem. Res..

[cit50] Kuncova-Kallio J., Kallio P. J. (2006). Conf. Proc. IEEE Eng. Med. Biol. Soc..

[cit51] Zhou J., Ellis A. V., Voelcker N. H. (2010). Electrophoresis.

[cit52] Merkel T. C., Bondar V. I., Nagai K., Freeman B. D., Pinnau I. (2000). J. Polym. Sci..

[cit53] Beebe D. J., Mensing G. A., Walker G. M. (2002). Annu. Rev. Biomed. Eng..

[cit54] Fiorini G. S., Chiu D. T. (2005). Biotechniques.

[cit55] Tsai J.-H., Lin L. (2002). Sens. Actuators, A.

[cit56] Teymoori M. M., Abbaspour-Sani E. (2005). Sens. Actuators, A.

[cit57] Byun C. K., Abi-Samra K., Cho Y. K., Takayama S. (2014). Electrophoresis.

[cit58] Huang P. H., Nama N., Mao Z., Li P., Rufo J., Chen Y., Xie Y., Wei C. H., Wang L., Huang T. J. (2014). Lab Chip.

[cit59] Terray A., Oakey J., Marr D. W. (2002). Science.

[cit60] Vestad T., Marr D. W. M., Oakey J. (2004). J. Micromech. Microeng..

[cit61] Chou H.-P., Unger M. A., Quake S. R. (2001). Biomed. Microdevices.

[cit62] Studer V., Pepin A., Chen Y., Ajdari A. (2004). Analyst.

[cit63] Lee C.-Y., Lee G.-B., Lin J.-L., Huang F.-C., Liao C.-S. (2005). J. Micromech. Microeng..

[cit64] Pollack M. G., Fair R. B., Shenderov A. D. (2000). Appl. Phys. Lett..

[cit65] Wang X., Cheng C., Wang S., Liu S. (2009). Microfluid. Nanofluid..

[cit66] Sung Kwon C., Hyejin M., Chang-Jin K. (2003). J. Microelectromech. Syst..

[cit67] Xia F., Tadigadapa S., Zhang Q. M. (2006). Sens. Actuators, A.

[cit68] Shen M., Dovat L., Gijs M. A. M. (2011). Sens. Actuators, B.

[cit69] Harmon M. E., Tang M., Frank C. W. (2003). Polymer.

[cit70] Berthier J., Dubois P., Clementz P., Claustre P., Peponnet C., Fouillet Y. (2007). Sens. Actuators, A.

[cit71] Jun D. H., Sim W. Y., Yang S. S. (2007). Sens. Actuators, A.

[cit72] Wong D. T. (2006). J. Am. Dent. Assoc..

[cit73] Upadhyaya S., Selvaganapathy P. R. (2010). Lab Chip.

[cit74] Gervais L., de Rooij N., Delamarche E. (2011). Adv. Mater..

[cit75] Hosokawa K., Sato K., Ichikawa N., Maeda M. (2004). Lab Chip.

[cit76] Sia S. K., Whitesides G. M. (2003). Electrophoresis.

[cit77] Bayraktar T., Pidugu S. B. (2006). Int. J. Heat Mass Transfer.

[cit78] Haeberle S., Zengerle R. (2007). Lab Chip.

[cit79] Young E. W., Beebe D. J. (2010). Chem. Soc. Rev..

[cit80] Ahn C. H. and Choi J.-W., in Springer Handbook of Nanotechnology, Springer Handbook, 2010, pp. 503–530, 10.1007/978-3-642-02525-9_18

[cit81] Su W., Gao X., Jiang L., Qin J. (2015). J. Chromatogr. A.

[cit82] Tripathi S., Varun Kumar Y. V. B., Prabhakar A., Joshi S. S., Agrawal A. (2015). J. Micromech. Microeng..

[cit83] Narayanamurthy V., Nagarajan S., Firus Khan A. a. Y., Samsuri F., Sridhar T. M. (2017). Anal. Methods.

[cit84] Guo J. H. W. and van der Wijngaart W., presented in part at the 20th International Conference on Miniaturized Systems for Chemistry and Life Sciences, Ireland, 2016

[cit85] Guo W., Hansson J., van der Wijngaart W. (2018). Microsyst. Nanoeng..

[cit86] Guo W. , Hansson J. and van der Wijngaart W., presented in part at the 2017 IEEE 30th International Conference on Micro Electro Mechanical Systems (MEMS), Las Vegas, NV, USA, Jan. 2017, 2017, vol. 22–26

[cit87] Microfluidics Market by Application (Genomics, Proteomics, Capillary Electrophoresis, IVD (POC, Clinical Diagnostics), Drug Delivery, Microreactor, Lab Tests), Component (Chips, Pump, Needle), Material (Polymer, Glass, Silicon) - Global Forecast to 2023, https://www.marketsandmarkets.com/Market-Reports/microfluidics-market-1305.html?gclid=CjwKCAiArK_fBRABEiwA0gOOc_eZ1aIgVylO7GQPYakcbg9F1lH6zEf-lw6jRwrmW0UWaEBWePRfCRoCw3MQAvD_BwE, 2019

[cit88] Gabrielse G., Fei X., Orozco L. A., Tjoelker R. L., Haas J., Kalinowsky H., Trainor T. A., Kells W. (1990). Phys. Rev. Lett..

[cit89] Yd S., Maroo S. C. (2016). Langmuir.

[cit90] Makhijani V. B., Reich A. J., Puntambekar A., Hong C., Ahn C. (2001). TechConnect Briefs.

[cit91] Walker G., Beebe D. J. (2002). Lab Chip.

[cit92] Walker G. M., Beebe D. J. (2002). Lab Chip.

[cit93] Chien-Fu C. , Shih-Chi K., Chin-Chou C. and Fan-Gang T., presented in part at the Sixteenth Annual International Conference on Micro Electro Mechanical Systems, 2003, MEMS-03 Kyoto, IEEE, Kyoto, Japan, Japan, 2003

[cit94] Ward T., Faivre M., Abkarian M., Stone H. A. (2005). Electrophoresis.

[cit95] Tan Y. C., Fisher J. S., Lee A. I., Cristini V., Lee A. P. (2004). Lab Chip.

[cit96] Yang Y., Liang Y. C. (2007). J. Power Sources.

[cit97] Berthier E., Beebe D. J. (2007). Lab Chip.

[cit98] Meyvantsson I., Warrick J. W., Hayes S., Skoien A., Beebe D. J. (2008). Lab Chip.

[cit99] Ju J., Park J. Y., Kim K. C., Kim H., Berthier E., Beebe D. J., Lee S.-H. (2008). J. Micromech. Microeng..

[cit100] Du Y., Shim J., Vidula M., Hancock M. J., Lo E., Chung B. G., Borenstein J. T., Khabiry M., Cropek D. M., Khademhosseini A. (2009). Lab Chip.

[cit101] Chen I. J., Eckstein E. C., Lindner E. (2009). Lab Chip.

[cit102] Resto P. J., Mogen B. J., Berthier E., Williams J. C. (2010). Lab Chip.

[cit103] Resto P. J., Mogen B., Wu F., Berthier E., Beebe D., Williams J. (2009). J. Visualized Exp..

[cit104] Chen I. J., Lindner E. (2009). Anal. Chem..

[cit105] McPherson A. L., Walker G. M. (2010). Microfluid. Nanofluid..

[cit106] Puccinelli J. P., Su X., Beebe D. J. (2010). JALA Charlottesv Va.

[cit107] Chung B. G., Choo J. (2010). Electrophoresis.

[cit108] Lin G., Lee A. P. (2010). Ann. N. Y. Acad. Sci..

[cit109] Berthier E., Warrick J., Casavant B., Beebe D. J. (2011). Lab Chip.

[cit110] Resto P. J., Berthier E., Beebe D. J., Williams J. C. (2012). Lab Chip.

[cit111] Kuo J. T., Li C., Meng E. (2014). Conf. Proc. IEEE Eng. Med. Biol. Soc..

[cit112] de Groot T. E., Veserat K. S., Berthier E., Beebe D. J., Theberge A. B. (2016). Lab Chip.

[cit113] Javadi K., Moezzi-Rafie H., Goodarzi-Ardakani V., Javadi A., Miller R. (2017). Colloids Surf., A.

[cit114] Iwai K. , Sochol R. D. and Lin L., presented in part at the 2011 IEEE 24th International Conference on Micro Electro Mechanical Systems, Cancun, Mexico, 2011

[cit115] Jönsson H. , Microfluidics for lab-on-a-chip applications, MSc thesis, Lund University, 2004

[cit116] Liu R. H., Stremler M. A., Sharp K. V., Olsen M. G., Santiago J. G., Adrian R. J., Aref H., Beebe D. J. (2000). J. Microelectromech. Syst..

[cit117] Ahn C. H. , Puntambekar A., Lee S. M., Cho H. J. and Hong C.-C., presented in part at the Micro Total Analysis Systems 2000, 2000

[cit118] Jeon N. L., Chiu D. T., Wargo C. J., Wu H., Choi I. S., Anderson J. R., Whitesides G. M. (2002). Biomed. Microdevices.

[cit119] Rush B. M., Dorfman K. D., Brenner H., Kim S. (2002). Ind. Eng. Chem. Res..

[cit120] Moorthy J., Beebe D. J. (2003). Lab Chip.

[cit121] Hu Y., Werner C., Li D. (2003). J. Fluids Eng..

[cit122] Chen C. o.-K., Cho C.-C. (2008). Chem. Eng. Sci..

[cit123] Jiang H., Weng X., Li D. (2010). Microfluid. Nanofluid..

[cit124] Hattori K., Sugiura S., Kanamori T. (2013). J. Lab. Autom..

[cit125] Davey N., Neild A. (2011). J. Colloid Interface Sci..

[cit126] Tice J. D., Desai A. V., Bassett T. A., Apblett C. A., Kenis P. J. A. (2014). RSC Adv..

[cit127] Iwai K., Shih K. C., Lin X., Brubaker T. A., Sochol R. D., Lin L. (2014). Lab Chip.

[cit128] Xu L., Lee H., Oh K. W. (2014). Microfluid. Nanofluid..

[cit129] Kokalj T., Park Y., Vencelj M., Jenko M., Lee L. P. (2014). Lab Chip.

[cit130] Jeong G. S., Oh J., Kim S. B., Dokmeci M. R., Bae H., Lee S. H., Khademhosseini A. (2014). Lab Chip.

[cit131] Tandon V., Kang W. S., Robbins T. A., Spencer A. J., Kim E. S., McKenna M. J., Kujawa S. G., Fiering J., Pararas E. E., Mescher M. J., Sewell W. F., Borenstein J. T. (2016). Lab Chip.

[cit132] Lee S., Kim H., Lee W., Kim J. (2018). Micro and Nano Systems Letters.

[cit133] Kim E. S., Gustenhoven E., Mescher M. J., Pararas E. E., Smith K. A., Spencer A. J., Tandon V., Borenstein J. T., Fiering J. (2014). Lab Chip.

[cit134] Satoh T., Narazaki G., Sugita R., Kobayashi H., Sugiura S., Kanamori T. (2016). Lab Chip.

[cit135] Zhang X., Wang X., Chen K., Cheng J., Xiang N., Ni Z. (2016). RSC Adv..

[cit136] Moon B. U., Abbasi N., Jones S. G., Hwang D. K., Tsai S. S. (2016). Anal. Chem..

[cit137] Ryu H., Choi K., Qu Y., Kwon T., Lee J. S., Han J. (2017). Anal. Chem..

[cit138] Nightingale A. M., Evans G. W., Xu P., Kim B. J., Hassan S. U., Niu X. (2017). Lab Chip.

[cit139] Dal Dosso F., Kokalj T., Belotserkovsky J., Spasic D., Lammertyn J. (2018). Biomed. Microdevices.

[cit140] Liu B., Li M., Tian B., Yang X., Yang J. (2018). Microfluid. Nanofluid..

[cit141] Boyd-Moss M., Baratchi S., Di Venere M., Khoshmanesh K. (2016). Lab Chip.

[cit142] Biscombe C. J. C. (2017). Angew. Chem., Int. Ed..

[cit143] Bruhn B. R., Schroeder T. B., Li S., Billeh Y. N., Wang K. W., Mayer M. (2014). PLoS One.

[cit144] Good B. T., Bowman C. N., Davis R. H. (2007). J. Colloid Interface Sci..

[cit145] Xu Z. R., Yang C. G., Liu C. H., Zhou Z., Fang J., Wang J. H. (2010). Talanta.

[cit146] Park J. Y., Kim S. K., Woo D. H., Lee E. J., Kim J. H., Lee S. H. (2009). Stem Cells.

[cit147] Paguirigan A. L., Beebe D. J. (2009). Integr. Biol..

[cit148] Park J. Y., Yoo S. J., Patel L., Lee S. H., Lee S. H. (2010). Biorheology.

[cit149] Kim S.-H., Kim K., Go M., Park J. Y. (2018). J. Micromech. Microeng..

[cit150] Herrlich S., Spieth S., Messner S., Zengerle R. (2012). Adv. Drug Delivery Rev..

[cit151] Juncker D., Schmid H., Drechsler U., Wolf H., Wolf M., Michel B., de Rooij N., Delamarche E. (2002). Anal. Chem..

[cit152] Hosokawa K., Maeda M. (2003). IEEJ Transactions on Sensors and Micromachines.

[cit153] Kim S.-J. , Shin Y. B., Lee D.-S., Yang H., Kim K., Park S. H. and Kim Y. T., presented in part at the 7th lnternational Conference on Miniaturized Chemical and Blochemlcal Analysts Systems, California USA, 2003

[cit154] Ichikawa N., Hosokawa K., Maeda R. (2004). J. Colloid Interface Sci..

[cit155] Zimmermann M., Schmid H., Hunziker P., Delamarche E. (2007). Lab Chip.

[cit156] Suk J. W., Cho J.-H. (2007). J. Micromech. Microeng..

[cit157] Zhu Y., Petkovic-Duran K. (2009). Microfluid. Nanofluid..

[cit158] Lynn N. S., Dandy D. S. (2009). Lab Chip.

[cit159] Gervais L., Delamarche E. (2009). Lab Chip.

[cit160] Mukhopadhyay S., Roy S. S., Mathur A., Tweedie M., McLaughlin J. A. (2010). J. Micromech. Microeng..

[cit161] Ng A. H., Uddayasankar U., Wheeler A. R. (2010). Anal. Bioanal. Chem..

[cit162] Kim Y. C., Kim S.-H., Kim D., Park S.-J., Park J.-K. (2010). Sens. Actuators, B.

[cit163] de Souza F. R., Alves G. L., Coltro W. K. (2012). Anal. Chem..

[cit164] Horiuchi T., Miura T., Iwasaki Y., Seyama M., Inoue S., Takahashi J., Haga T., Tamechika E. (2012). Sensors.

[cit165] Kim S. J., Paczesny S., Takayama S., Kurabayashi K. (2013). Lab Chip.

[cit166] Kistrup K., Poulsen C. E., Østergaard P. F., Haugshøj K. B., Taboryski R., Wolff A., Hansen M. F. (2014). J. Micromech. Microeng..

[cit167] Berthier J., Brakke K. A., Gosselin D., Bourdat A. G., Nonglaton G., Villard N., Laffite G., Boizot F., Costa G., Delapierre G. (2014). Microfluid. Nanofluid..

[cit168] Madadi H., Casals-Terre J., Mohammadi M. (2015). Biofabrication.

[cit169] Nie C., Frijns A. J., Mandamparambil R., den Toonder J. M. (2015). Biomed. Microdevices.

[cit170] Mukhopadhyay S., Banerjee J. P., Roy S. S., Metya S. K., Tweedie M., McLaughlin J. A. (2017). Surf. Rev. Lett..

[cit171] Huet M., Cubizolles M., Buhot A. (2017). Biosens. Bioelectron..

[cit172] Bunge F., van den Driesche S., Vellekoop M. J. (2016). Microfluid. Nanofluid..

[cit173] Wu T., Luo Z., Ding W., Cheng Z., He L. (2017). Microfluid. Nanofluid..

[cit174] Zhai Y., Wang A., Koh D., Schneider P., Oh K. W. (2018). Lab Chip.

[cit175] Xie J., Su H., Liao J., Liu J. (2017). Microfluid. Nanofluid..

[cit176] Vasilakis N., Papadimitriou K. I., Morgan H., Prodromakis T. (2017). Microfluid. Nanofluid..

[cit177] Akyazi T., Gil-González N., Basabe-Desmonts L., Castaño E., Morant-Miñana M. C., Benito-Lopez F. (2017). Sens. Actuators, B.

[cit178] Frimat J.-P., Schurink B., Luttge R. (2017). J. Vac. Sci. Technol., B: Nanotechnol. Microelectron.: Mater., Process., Meas., Phenom..

[cit179] Mei L., Jin M., Xie S., Yan Z., Wang X., Zhou G., van den Berg A., Shui L. (2018). Lab Chip.

[cit180] Kim B., Oh S., Shin S., Yim S. G., Yang S. Y., Hahn Y. K., Choi S. (2018). Anal. Chem..

[cit181] Moonen E., Luttge R., Frimat J.-P. (2018). Microelectron. Eng..

[cit182] Tachibana H., Saito M., Tsuji K., Yamanaka K., Hoa L. Q., Tamiya E. (2015). Sens. Actuators, B.

[cit183] Aeinehvand M. M., Ibrahim F., Harun S. W., Al-Faqheri W., Thio T. H., Kazemzadeh A., Madou M. (2014). Lab Chip.

[cit184] Olanrewaju A., Beaugrand M., Yafia M., Juncker D. (2018). Lab Chip.

[cit185] Mäki A.-J., Hemmilä S., Hirvonen J., Girish N. N., Kreutzer J., Hyttinen J., Kallio P. (2015). J. Fluids Eng..

[cit186] Cho B. S., Schuster T. G., Zhu X., Chang D., Smith G. D., Takayama S. (2003). Anal. Chem..

[cit187] Suh R. S., Phadke N., Ohl D. A., Takayama S., Smith G. D. (2003). Hum. Reprod. Update.

[cit188] Yamada H., Yoshida Y., Terada N. (2005). Jpn. J. Appl. Phys..

[cit189] Huh D., Bahng J. H., Ling Y., Wei H. H., Kripfgans O. D., Fowlkes J. B., Grotberg J. B., Takayama S. (2007). Anal. Chem..

[cit190] Lee P. J., Ghorashian N., Gaige T. A., Hung P. J. (2007). JALA Charlottesv Va.

[cit191] Zhang K., Liang Q., Ma S., He T., Ai X., Hu P., Wang Y., Luo G. (2010). Microfluid. Nanofluid..

[cit192] Sung J. H., Kam C., Shuler M. L. (2010). Lab Chip.

[cit193] Esch M. B., Prot J. M., Wang Y. I., Miller P., Llamas-Vidales J. R., Naughton B. A., Applegate D. R., Shuler M. L. (2015). Lab Chip.

[cit194] Jin D., Deng B., Li J. X., Cai W., Tu L., Chen J., Wu Q., Wang W. H. (2015). Biomicrofluidics.

[cit195] James J. , Sushmitha M., Premkumar R., Narayanamurthy V. and Kalpana R., presented in part at the 2017 International conference on Microelectronic Devices, Circuits and Systems (ICMDCS), Vellore, India, 2017

[cit196] Narayanamurthy V., Lee T., Khan A. a., Samsuri F., Mohamed K., Hamzah H., Baharom M. (2018). Fluids.

[cit197] Kim S.-J. , Zhu X. and Takayama S., in Microtechnology for Cell Manipulation and Sorting, Springer, 2017, pp. 175–192, 10.1007/978-3-319-44139-9_6

[cit198] Seo D.-b., Agca Y., Feng Z. C., Critser J. K. (2007). Microfluid. Nanofluid..

[cit199] Knowlton S. M., Sadasivam M., Tasoglu S. (2015). Trends Biotechnol..

[cit200] Shah P. J. , Dimaki M. and Svendsen W. E., presented in part at the TRANSDUCERS 2009 - 2009 International Solid-State Sensors, Actuators and Microsystems Conference, Denver, CO, USA, 2009

[cit201] Benefits of Water Therapy, https://www.friendshiphaven.org/benefits-of-water-therapy/

[cit202] Weigl B. H. , Bardell R., Schulte T. and Williams C., presented in part at the Micro Total Analysis Systems 2000, 2000

[cit203] Weigl B. H., Bardell R. L., Kesler N., Morris C. J. (2014). Fresenius. J. Anal. Chem..

[cit204] Marimuthu M., Kim S. (2013). Anal. Biochem..

[cit205] Komeya M., Hayashi K., Nakamura H., Yamanaka H., Sanjo H., Kojima K., Sato T., Yao M., Kimura H., Fujii T., Ogawa T. (2017). Sci. Rep..

[cit206] Wu W. (2018). Analyst.

[cit207] Song Q., Sun J., Mu Y., Xu Y., Zhu Q., Jin Q. (2018). Sens. Actuators, B.

[cit208] Monahan J., Gewirth A. A., Nuzzo R. G. (2001). Anal. Chem..

[cit209] Hosokawa K., Omata M., Sato K., Maeda M. (2006). Lab Chip.

[cit210] Dimov I. K., Basabe-Desmonts L., Garcia-Cordero J. L., Ross B. M., Park Y., Ricco A. J., Lee L. P. (2011). Lab Chip.

[cit211] Liang D. Y., Tentori A. M., Dimov I. K., Lee L. P. (2011). Biomicrofluidics.

[cit212] Li G., Luo Y., Chen Q., Liao L., Zhao J. (2012). Biomicrofluidics.

[cit213] Li C. G., Dangol M., Lee C. Y., Jang M., Jung H. (2015). Lab Chip.

[cit214] Xu L., Lee H., Jetta D., Oh K. W. (2015). Lab Chip.

[cit215] Li C., Xu J., Ma B. (2014). Microfluid. Nanofluid..

[cit216] Agustini D., Bergamini M. F., Marcolino-Junior L. H. (2017). Anal. Chim. Acta.

[cit217] Liu B., Koh D., Wang A., Schneider P., Oh K. W. (2017). Microsyst. Technol..

[cit218] Li Y., Jiang Y., Wang K., Wu W. (2018). Anal. Chem..

[cit219] Liu Y., Li G. (2018). Sci. Rep..

[cit220] Warrick J., Meyvantsson I., Ju J., Beebe D. J. (2007). Lab Chip.

[cit221] Verneuil E., Buguin A., Silberzan P. (2004). Europhys. Lett..

[cit222] Randall G. C., Doyle P. S. (2005). Proc. Natl. Acad. Sci. U. S. A..

[cit223] Eddings M. A., Gale B. K. (2006). J. Micromech. Microeng..

[cit224] Weibel D. B., Siegel A. C., Lee A., George A. H., Whitesides G. M. (2007). Lab Chip.

[cit225] Johnson M., Liddiard G., Eddings M., Gale B. (2009). J. Micromech. Microeng..

[cit226] Chiu D. T., deMello A. J., Di Carlo D., Doyle P. S., Hansen C., Maceiczyk R. M., Wootton R. C. R. (2017). Chem.

[cit227] Leslie D. C., Easley C. J., Seker E., Karlinsey J. M., Utz M., Begley M. R., Landers J. P. (2009). Nat. Phys..

[cit228] Atalay Y. T., Vermeir S., Witters D., Vergauwe N., Verbruggen B., Verboven P., Nicolaï B. M., Lammertyn J. (2011). Trends Food Sci. Technol..

[cit229] Bélanger M.-C., Marois Y. (2001). J. Biomed. Mater. Res..

[cit230] Kachel S., Zhou Y., Scharfer P., Vrancic C., Petrich W., Schabel W. (2014). Lab Chip.

[cit231] Hitzbleck M., Delamarche E. (2013). Micromachines.

[cit232] Shah P., Zhu X., Chen C., Hu Y., Li C. Z. (2014). Biomed. Microdevices.

[cit233] Global tuberculosis report 2014, http://www.who.int/iris/handle/10665/137094, accessed March, 2019

[cit234] McNerney R. (2015). Diagnostics.

[cit235] W. H. Organization , World health statistics 2016: monitoring health for the SDGs sustainable development goals, World Health Organization, 2016

